# Codon-by-Codon Modulation of Translational Speed and Accuracy Via mRNA Folding

**DOI:** 10.1371/journal.pbio.1001910

**Published:** 2014-07-22

**Authors:** Jian-Rong Yang, Xiaoshu Chen, Jianzhi Zhang

**Affiliations:** Department of Ecology and Evolutionary Biology, University of Michigan, Ann Arbor, Michigan, United States of America; Fred Hutchinson Cancer Research Center, United States of America

## Abstract

Rapid cell growth demands fast protein translational elongation to alleviate ribosome shortage. However, speedy elongation undermines translational accuracy because of a mechanistic tradeoff. Here we provide genomic evidence in budding yeast and mouse embryonic stem cells that the efficiency–accuracy conflict is alleviated by slowing down the elongation at structurally or functionally important residues to ensure their translational accuracies while sacrificing the accuracy for speed at other residues. Our computational analysis in yeast with codon resolution suggests that mRNA secondary structures serve as elongation brakes to control the speed and hence the fidelity of protein translation. The position-specific effect of mRNA folding on translational accuracy is further demonstrated experimentally by swapping synonymous codons in a yeast transgene. Our findings explain why highly expressed genes tend to have strong mRNA folding, slow translational elongation, and conserved protein sequences. The exquisite codon-by-codon translational modulation uncovered here is a testament to the power of natural selection in mitigating efficiency–accuracy conflicts, which are prevalent in biology.

## Abstract 2

Secondary structure in mRNAs modulates the speed of protein synthesis codon-by-codon to improve accuracy at important sites while ensuring high speed elsewhere.

## Author Summary

Protein synthesis by ribosomal translation is a vital cellular process, but our understanding of its regulation has been poor. Because the number of ribosomes in the cell is limited, rapid growth relies on fast translational elongation. The accuracy of translation must also be maintained, and in an ideal scenario, both speed and accuracy should be maximized to sustain rapid and productive growth. However, existing data suggest a tradeoff between speed and accuracy, making it impossible to simultaneously maximize both. A potential solution is slowing the elongation at functionally or structurally important sites to ensure their translational accuracies, while sacrificing accuracy for speed at other sites. Here, we show that budding yeast and mouse embryonic stem cells indeed use this strategy. We discover that a codon-by-codon adaptive modulation of translational elongation is accomplished by mRNA secondary structures, which serve as brakes to control the elongation speed and hence translational fidelity. Our findings explain why highly expressed genes tend to have strong mRNA folding, slow translational elongation, and conserved protein sequences. The exquisite translational modulation reflects the power of natural selection in mitigating efficiency–accuracy conflicts, and our study offers a general framework for analyzing similar conflicts, which are widespread in biology.

## Introduction

Rapid cell growth demands expeditious protein synthesis, which requires a large number of ribosomes. Because ribosomes are limited during rapid cell growth [1], fast translational elongation is desired to minimize ribosome sequestration and alleviate ribosome shortage [2]–[4]. However, several lines of evidence suggest that speedy elongation undermines translational accuracy when other cellular factors such as tRNA concentrations are kept constant [5]. For example, mutations in *Escherichia coli* that increase the elongation speed decrease translational fidelity, and vice versa [6]. Similarly, adjusting the Mg^2+^ concentration during *in vitro* protein synthesis has opposite effects on the elongation speed and translational accuracy (see Figure S1A for a potential mechanistic explanation of the tradeoff between the speed and accuracy) [7]. Translational errors are harmful, because of the material and energy waste in synthesizing dysfunctional proteins and the increased risk of deleterious protein misfolding [8]–[10] and misinteraction [11].

Given the tradeoff between translational accuracy and elongation speed, a fascinating question is how cells respond if the objective is rapid growth. An obvious strategy is sacrificing speed for accuracy at residues that require accurate translation, while forgoing accuracy for speed at residues where errors are tolerable. Here we investigate whether cells indeed use this strategy and the potential mechanism allowing for the modulation of elongation speed and accuracy codon by codon. The study is made possible by the recent development of the ribosome profiling technique [12], which allows estimating elongation speed with codon resolution. In fact, analyses of ribosome profiling data have identified several factors that impact the elongation speed, including, for example, codon usage relative to tRNA concentrations [3], mRNA secondary structure [13], and positively charged amino acids [14] in eukaryotes and anti–Shine-Dalgarno sequence in prokaryotes [15]. Here we focus on the budding yeast *Saccharomyces cerevisiae* unless otherwise noted, because of the availability of various datasets in this model eukaryote that are necessary for our analysis.

## Results

### Conserved Yeast Genes Tend to Have Relatively Slow Translational Elongations

We first used ribosome profiling data to estimate the ribosome density at each codon of each mRNA, which is the relative number of ribosomes whose aminoacyl (A) site is occupied by the codon at a given moment [3]. Assuming negligible ribosome dropoff [3],[16] and homeostasis of cellular protein abundance, we further estimated the translational initiation rates from genomic measurements of mRNA expression levels [12], protein abundances [17], and protein degradation rates (see Materials and Methods) [18]. The relative elongation speed of a codon in an mRNA is the translational initiation rate of the mRNA divided by the ribosome density of the codon [3]. We removed the first 50 codons of each coding sequence from our elongation speed analysis to avoid the potential interferences from the reported 5′ elongation “ramp” [19] and factors related to translational initiation [4],[20].

**Figure 1:**
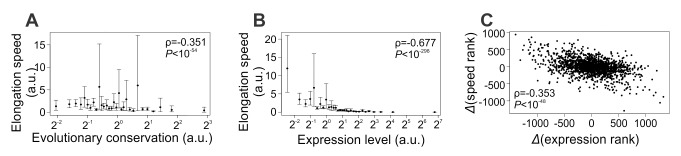
Slower translational elongation of yeast genes with higher demands for translational accuracy. (A) Average elongation speed for a gene decreases as the mean evolutionary conservation of the gene at the protein sequence level increases. The 1,862 yeast genes analyzed are grouped into 30 equal-sized bins. Error bars indicate the 95% confidence interval of the mean, estimated by bootstrapping the genes 1,000 times. The plot is shown in a log scale on the x axis because evolutionary conservation is calculated by the inverse of evolutionary rate and hence can be very large for proteins with very low rates of evolution. Spearman's rank correlation of the original unbinned data is shown. Note that the rank correlation does not depend on the scale used in the plot. (B) Average elongation speed for a gene decreases as the expression level of the gene increases. The 2,237 yeast genes analyzed are grouped into 30 equal-sized bins. Error bars indicate the 95% confidence interval of the mean, estimated by bootstrapping the genes 1,000 times. The plot is shown in a log scale on the x axis because the frequency distribution of gene expression level is known to follow a power law. Spearman's rank correlation of the original unbinned data is shown. Note that the rank correlation does not depend on the scale used in the plot. (C) Gene expression rank changes due to an environmental shift from a rich medium to an amino acid starvation medium are negatively correlated with changes in the rank of translational elongation speed. The 1,653 yeast genes that have relevant information are each depicted by a dot.

The evolutionary conservation of a residue among orthologous proteins (i.e., the inverse of its evolutionary rate) is a proxy for its structural and/or functional importance [21] and hence the requirement for translational accuracy [8]. Similarly, the average conservation of all residues of a protein measures the average requirement for its translational accuracy. Hence, our hypothesized solution to the tradeoff between translational fidelity and elongation speed predicts that the more conserved a gene is, the slower its translational elongation is. Indeed, the average elongation speed of an mRNA is negatively correlated with the average evolutionary conservation of its protein sequence estimated by comparing with orthologs from five other fungal species (Figure 1A; see Materials and Methods for the calculation of averages), suggesting that our hypothesized solution is used by yeast cells at least at the gene level. While the above ribosome profiling dataset was generated from a strain with the S288C background [12], the same pattern was observed when two additional yeast ribosome profiling datasets, generated in two other strains both of the SK1 background [22], were analyzed (Figure S1B and D).

### Theoretical and Empirical Evidence for Decelerated Elongation of Highly Expressed Genes

The expression level of a gene (i.e., its cellular mRNA concentration) is another potential predictor of the requirement for translational accuracy, because the fitness cost of mistranslation increases with the number of translational errors, which is proportional to the amount of protein synthesis and hence mRNA concentration [8]. Nevertheless, the selective pressure for fast elongation to lessen ribosome sequestration also intensifies as the expression level of a gene rises, because a given increase in elongation speed reduces ribosome sequestration more when occurring to a highly expressed gene than to a lowly expressed gene. To predict the outcome of these competing demands, we built a mathematical model that estimates the fitness impact of translational speed and accuracy (see Materials and Methods).

**Figure 2:**
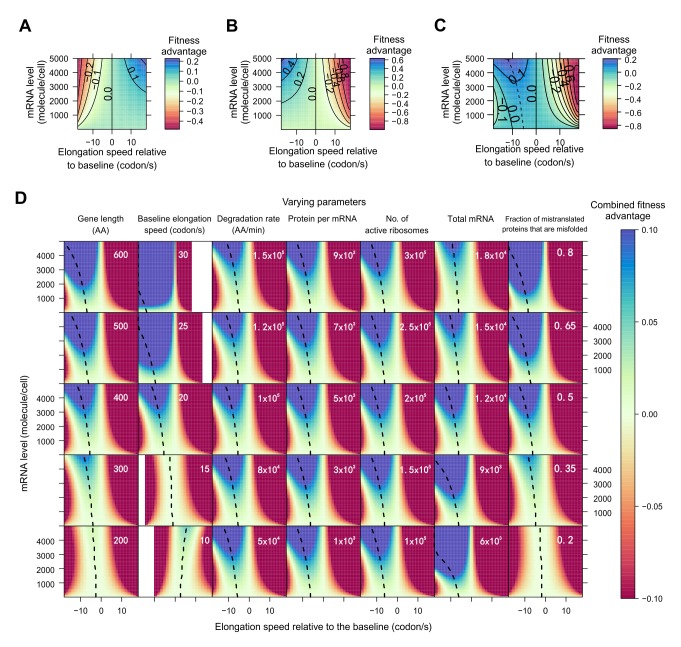
Model prediction of the fitness effect as a function of the relative elongation speed and expression level of a focal gene. (A) Fitness effect of an increase in elongation speed that mitigates ribosome sequestration. The fitness advantage is shown as a function of elongation speed (relative to the baseline of 20 codons per second) and gene expression level of the focal gene. The magnitude of fitness advantage is shown by different colors, and each solid contour line shows the combinations of gene expression level and elongation speed that result in the same fitness effect. (B) Fitness effect of an increase in elongation speed that reduces translational fidelity. (C) Combined fitness effect of an increase in elongation speed that mitigates ribosome sequestration but also reduces translational fidelity. The dotted line depicts the speed change that results in the biggest fitness increase for a gene with a given expression level. (D) Combined fitness effect of an increase in elongation speed when each of seven parameters in the model varies. Each column of plots has one varying parameter (indicated at the top of the column), whereas the other six parameters remain constant. The value of the varying parameter is indicated in each plot, whereas the values of the other six parameters are indicated in the plots of the middle row. White space in heat maps indicates undefined regions due to either negative elongation speeds or higher translation error rates than allowed by the model. Total mRNA refers to the total number of mRNA molecules per cell.

The first part of our model considers the benefit of reducing ribosome sequestration by accelerated elongation. The model assumes equilibrium in cellular protein concentration for each gene, which is achieved by a balance of protein synthesis, degradation, and dilution due to cell division. We parameterized the model with the best estimates from the literature (see Materials and Methods) and calculated the fitness advantage (*s_v_*) due to a predefined change (*Δν*) in elongation speed from a baseline for gene *g*. This model showed that, given *Δν*, the absolute value of *s_v_* is greater when *Δν* occurs to a highly expressed gene than to a lowly expressed gene (Figure 2A). Furthermore, given the expression level, the fitness advantage does not increase linearly with *Δν*, but shows a diminishing return, evident from the increasing distances between the contour lines when *Δν* increases (Figure 2A). This phenomenon is expected, because as *Δν* in gene *g* increases, ribosomes spend a larger fraction of time on genes other than *g*, effectively reducing the benefit of the increased elongation speed in *g*.

The second part of our model addresses the fitness cost of mistranslation caused by accelerated elongation. We extrapolated the quantitative relationship between elongation speed and accuracy from experimentally determined tRNA selection reaction rates [7]. The fitness cost of mistranslation is estimated by assuming that protein molecules containing errors tend to misfold [8],[10] and by using the recently measured fitness cost of protein misfolding in yeast [9]. Similar to the first part of the model, we estimated the fitness effect (*s*_t_) of translational errors under different *Δν* and expression levels. The model showed that, given *Δν*, the absolute value of *s*_t_ is greater when *Δν* occurs to a highly expressed gene than to a lowly expressed gene (Figure 2B).

We then combined the two parts of our model (*s* = *s_v_*+*s*_t_) to estimate the net gain in fitness due to changes in elongation speed (Figure 2C). We found that the optimal *Δν* (i.e., the *Δν* maximizing the fitness) is −12.2 and −5.3 codons per second for genes with the highest (5,000 mRNA molecules/cell) and lowest (1) expressions considered, respectively. More importantly, our model predicts a negative correlation between the expression level of a gene and its optimal *Δν* (the dotted line in Figure 2C). This prediction appears to be robust to almost all variations of the parameters in the model (Figure 2D). It is worth pointing out here that, due to the complexity of modeling, we did not consider the loss-of-function effect of translational errors in our model. Because such errors are expected to have bigger effects on highly expressed genes than on lowly expressed genes [23],[24], they would further reduce the optimal elongation speed for highly expressed genes, but would have a minimal impact on lowly expressed genes.

The prediction of our model is empirically supported. Specifically, analysis of each of the three yeast ribosome profiling datasets reveals a significantly negative correlation between mRNA concentration and elongation speed (Figure 1B, Figure S1C and E). Hence, both our model and the empirical data show that, for highly expressed genes, the demand for translational fidelity trumps that for fast elongation. Furthermore, the partial correlation between expression level and elongation speed remains significant after the control of evolutionary conservation (Spearman's ρ = −0.606, *p*<10^−236^); so does the partial correlation between evolutionary conservation and elongation speed after the control of expression level (ρ = −0.112, *p*<10^−5^).

While all of the above analyses used the ribosome profiling data and gene expression data from rich media, the corresponding data generated from a starvation condition is available for the strain with the S288C background [12]. If the elongation speed of a gene can be regulated across environments (see Discussion), our model (Figure 2C) would predict a lower speed in the environment where the gene expression is higher. Supporting this prediction, we observed a negative correlation between a gene's between-environment difference in the rank of expression level and that of elongation speed (Figure 1C). As a negative control, we repeated the above analysis using data from two replicated experiments under the rich media [12]. Indeed, the correlation observed in Figure 1C now vanishes (Figure S1F; see also Materials and Methods), confirming that the original correlation in Figure 1C is genuine.

### Evidence from the Ribosome Run-Off Experiment in Mouse Embryonic Stem Cells

**Figure 3:**
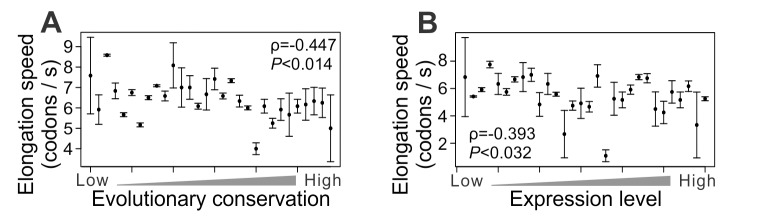
Slower translational elongations of genes with higher demands for translational accuracy, on the basis of ribosome run-off experiment in mouse embryonic stem cells. (A) Average elongation speed for a meta-gene representing a group of genes decreases as the mean evolutionary conservation of the group at the protein sequence level increases. The 1,037 mouse genes analyzed are divided into 30 equal-sized groups, each represented by a meta-gene. Error bar indicates one standard error. (B) Average elongation speed for a meta-gene representing a group of genes decreases as the expression level of the gene group increases. The 381 genes analyzed are divided into 30 equal-sized groups, each represented by a meta-gene. Error bar indicates one standard error.

Mouse embryonic stem cells were recently subjected to a ribosome run-off experiment, which directly estimates the mean elongation speed for a segment of mRNA without the need to know the translational initiation rate [25]. Because of the design and the limited resolution of the experiment [25], we estimated the elongation speed for meta-genes representing groups of genes rather than individual genes (see Materials and Methods). We found the average elongation speed of a gene group to be negatively correlated with both its mean protein sequence conservation (Figure 3A) and mean expression level (Figure 3B), suggesting that both the conflict between translational speed and accuracy and its resolution are similar between unicellular and multicellular eukaryotes. These findings also demonstrate the robustness of our results to different experimental approaches and analyses (see Materials and Methods).

### Within-Gene Analysis Reveals Codon-by-Codon Modulation of Elongation Speed and Accuracy

**Figure 4:**
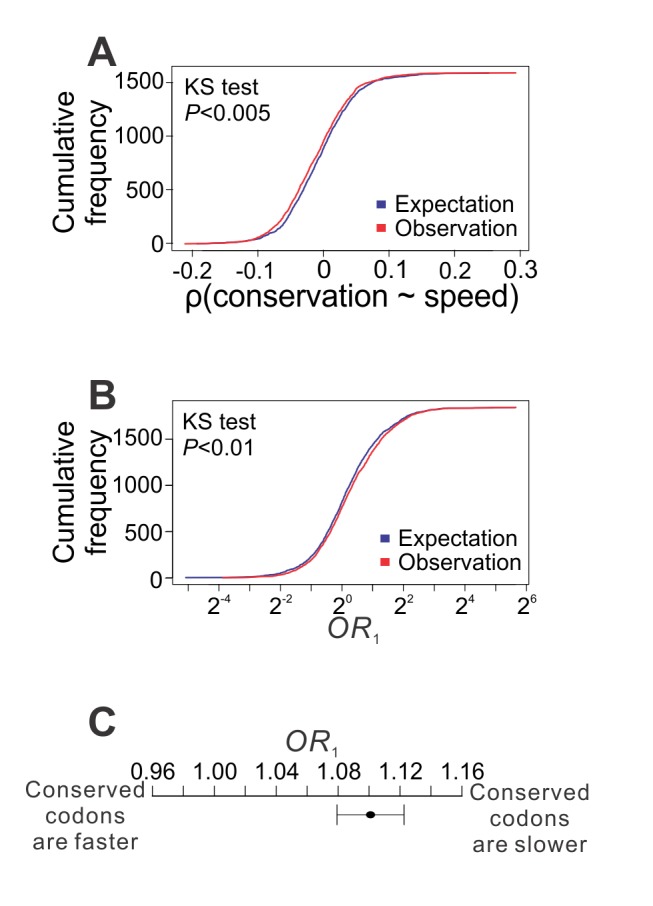
Within individual yeast genes, codons with higher demands for translational accuracy have slower elongations. A total of 1,843 genes are used. (A) Cumulative frequency distributions for the observed and randomly expected within-gene rank correlations between the evolutionary conservation of the encoded amino acid of a codon and its elongation speed. KS test, Kolmogorov–Smirnov test of the equality of the two distributions. Only 1,590 genes are used here because correlation cannot be calculated for the other 254 genes due to the lack of variation (or having too few sites with necessary data) in either evolutionary conservation or elongation speed. (B) Cumulative frequency distributions for the observed and randomly expected odds ratio *OR*_1_, which measures the enrichment of slow-elongation codons at evolutionary conserved residues within a gene. (C) Combined *OR*_1_ for all genes examined, by the MH procedure. The combined *OR*_1_ significantly exceeds 1 (*p*<10^−7^, MH test). Error bar indicates one standard error, estimated by bootstrapping the genes 1,000 times.

After examining the elongation speed variation among genes, we analyzed it among codons within each yeast gene. Because all codons within a gene share the same translational initiation rate, this analysis is uninfluenced by potential errors in the initiation rate estimation. We first focused on the ribosome profiling data from the strain with the S288C background [12]. Among the 1,590 genes with necessary information, the rank correlation between the elongation speed at a codon and the evolutionary conservation of the corresponding amino acid residue encoded by the codon is negative for 854 genes, significantly more than the random expectation of 1,590/2 = 795 (*p*<0.002, binomial test). The ribosome profiling data from the two strains of the SK1 background also show similar results (strain A14201, 1,202 out of 1,985 genes, *p*<2×10^−21^; strain gb15, 1,207 out of 1,999 genes, *p*<5×10^−21^). We also randomly shuffled the elongation speeds of all codons within a gene and calculated the correlation between speed and conservation. Compared with the correlations calculated from the randomly shuffled data, the real correlations are skewed toward negative values (Figure 4A), as predicted by our hypothesis. To further evaluate the relationship between speed and conservation within a gene, we constructed a 2×2 table by classifying each codon in the gene into one of four categories based on its elongation speed and evolutionary conservation, and calculated an odds ratio (*OR*_1_) from the table (see Materials and Methods). The greater the *OR*_1_ (relative to 1), the stronger the support for our hypothesized strategy. We similarly generated a randomly expected *OR*_1_ by shuffling the elongation speeds among codons within the gene. We found that the real *OR*_1_ values are significantly skewed toward larger values when compared with their random expectations (Figure 4B). Using the Mantel–Haenszel (MH) procedure to combine the information from all genes, we found the overall *OR*_1_ to exceed 1 significantly (Figure 4C). Similar patterns as shown in Figure 4 were observed when the yeast ribosome profiling data from two other strains [22] were analyzed (Figure S2). The relatively small deviations from expectations for the correlation shown in Figure 4A and *OR*_1_ shown in Figure 4B are not unexpected, because these analyses were carried out at the codon level, where stochasticity in the ribosome profiling data is substantial due to the small number of reads per codon. To better gauge the effect size, we calculated the ratio in ribosome density between conserved codons (i.e., with invariant amino acids among the six fungal species examined) and nonconserved codons within each gene. In the 100 genes with the highest expressions (which are expected to have the largest numbers of reads), the median and mean of this ratio are 1.07 and 1.45, respectively, corresponding to a 7% and 30% deceleration of elongation, respectively, for conserved codons compared with nonconserved codons (see Text S13). Collectively, these results support our hypothesis that, within a gene, the translational elongation of a codon is slower when the demand for accuracy is higher.

### The Mechanism of Modulating the Tradeoff Between Elongation Speed and Accuracy

**Figure 5:**
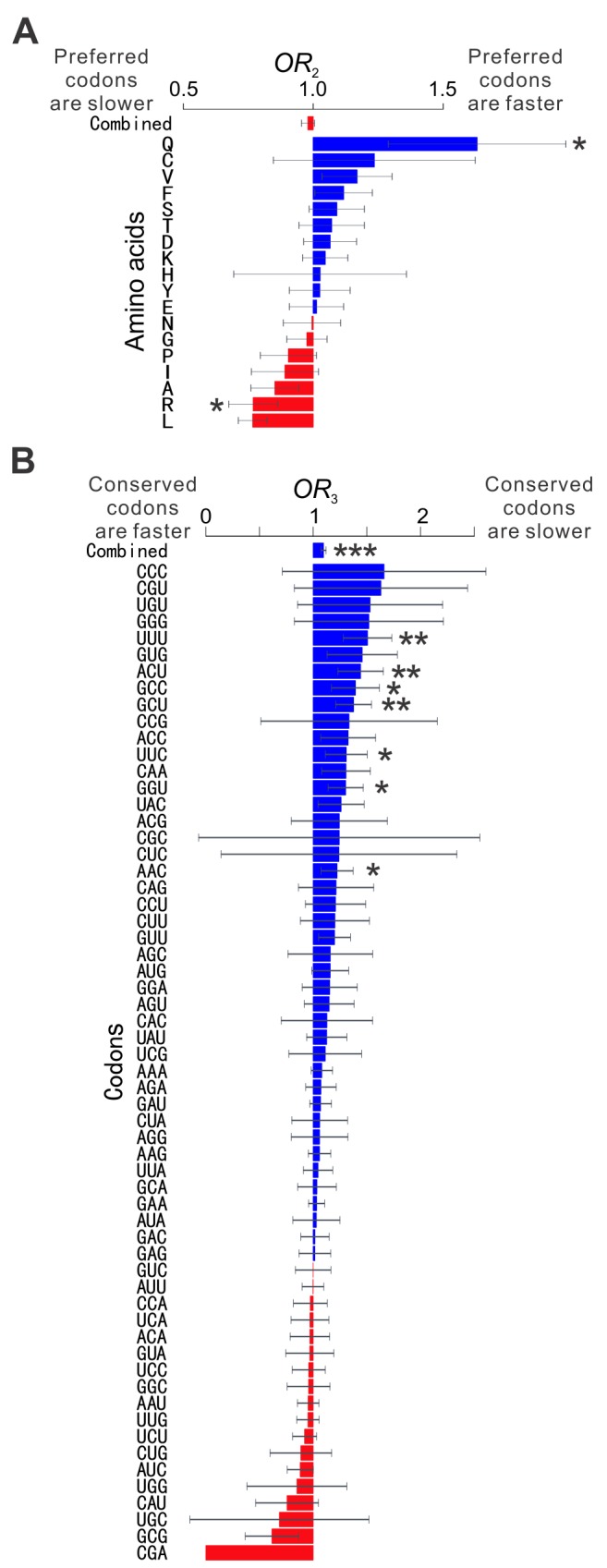
Among residues encoded by the same codon within the same gene, those with higher demands for translational accuracy have lower elongation speeds. A total of 1,843 genes are used. (A) Synonymous codon usage does not predict elongation speed. For each amino acid, *OR*_2_ is calculated for each gene and then combined across genes by the MH procedure. *OR*_2_>1 indicates that preferred codons are translated faster than unpreferred codons, and vice versa. The combined *OR*_2_ from all amino acids is not significantly different from 1 (*p*>0.2). (B) Among residues encoded by the same codon in the same gene, those that are more conserved are translated more slowly. For each codon, *OR*_3_ is calculated for each gene and then combined across genes by the MH procedure. *OR*_3_>1 indicates that conserved residues encoded by a codon are translated more slowly than unconserved ones encoded by the same codon, and vice versa. The combined *OR*_3_ from all codons is significantly greater than 1 (*p*<10^−5^). For both panels, error bar indicates one standard error, estimated by bootstrapping the genes 1,000 times. The standard error of *OR*_3_ for CGA cannot be estimated because CGA with relevant information occurred in only one gene. Nominal *p* values from the MH test are indicated by asterisks. * *p*<0.05; ** *p*<0.01; *** *p*<0.001.

What is the molecular mechanism underlying the codon-level modulation of the tradeoff between elongation speed and translational accuracy? A potential answer is synonymous codon usage bias (CUB). It has long been assumed that preferentially used synonymous codons are translated faster than unpreferred codons due to differences in the concentration of their cognate tRNAs [26]–[28]. Recent studies in *E. coli*, yeast, and mouse with ribosome profiling or run-off experiments, however, found similar elongation speeds for synonymous codons [3],[15],[25]. Hence, despite that CUB is known to impact translational accuracy [8],[29], CUB is unlikely to modulate the speed–accuracy conflict. To confirm this, for each of the 18 amino acids encoded by more than one codon, we used an odds ratio (*OR*_2_; see Materials and Methods) to measure, within each gene, the relationship between the preference and elongation speed of its synonymous codons. *OR*_2_>1 indicates that preferred codons are translated faster than unpreferred codons, and vice versa. We then combined such information from all genes with available information, by the MH procedure. For the strain of the S288C background, 11 amino acids show *OR*_2_>1 (Figure 5A), not significantly more than the random expectation of 9 (*p*>0.20, binomial test). The overall *OR*_2_ combined from 18 amino acids by the MH procedure is 0.98, not significantly different from 1 (*p* = 0.215; Figure 5A). The same analysis was conducted for the other two yeast strains and similar patterns were observed (Figure S3A and C). These results demonstrate that the within-gene variation in elongation speed among all codons of an amino acid is not attributable to differences among synonymous codons, suggesting that even the same codon would have different elongation speeds according to different requirements for translational accuracy at different positions of a protein. To verify this prediction, we defined *OR*_3_, which is the same as *OR*_1_ except that it is calculated for each of the 61 sense codons (see Materials and Methods). That is, for a given codon in a given gene, *OR*_3_>1 indicates that evolutionary conservation predicts slow translation, and vice versa. Because of the lack of data for one of the codons, we calculated *OR*_3_ for each of the remaining 60 sense codons in each gene and then statistically combined them across genes. Because *OR*_3_ is calculated separately for each codon, it has a relatively large standard error, especially for codons that are rarely used (Figure 5B). Nonetheless, 42 codons show *OR*_3_>1, significantly more than the random expectation of 30 (*p* = 0.001, binomial test). There are seven codons whose *OR*_3_>1 significantly, whereas no codon has *OR*_3_<1 significantly (Figure 5B). When combined for all codons, *OR*_3_ significantly exceeds 1 (Figure 5B). As anticipated from the result on *OR*_1_ (Figure 4B), the deviation of *OR*_3_ from 1 is relatively small. However, the actual size of effect on translational accuracy by slowed elongation would be better estimated by controlled experiments for individual codons (see below). Notwithstanding, the above observations from the strain of the S288C background, along with similar results from the two strains with the SK1 background (Figure S3B and D), confirm our prediction and suggest that the regulation of the elongation speed of a codon relies on information beyond the identity of the codon.

**Figure 6:**
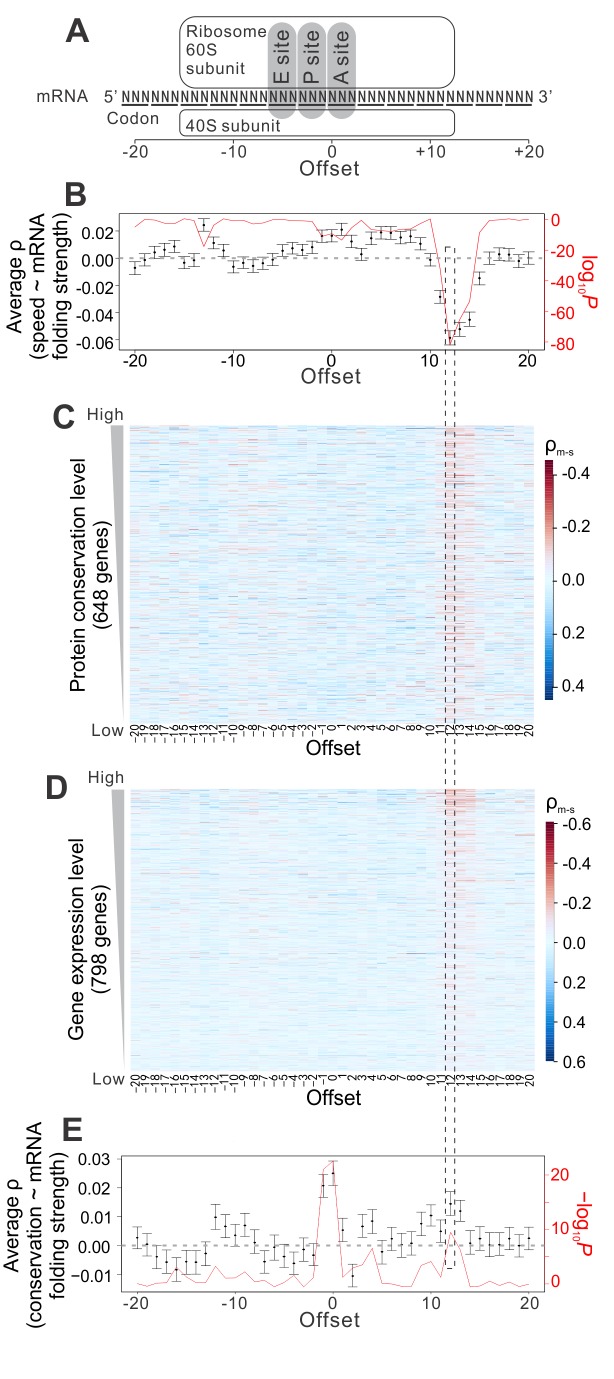
Messenger RNA folding serves as elongation brakes to modulate the speed and accuracy of protein translation in yeast. (A) Schematic diagram of a translating ribosome. The codon being decoded is at the ribosome A site. (B) Rank correlation (black dots) between the elongation speed (measured at the ribosome A site) and mRNA folding strength at different offsets. The correlations are calculated among codons within each gene; the 1,232 within-gene correlations are then averaged. Error bar indicates 95% confidence interval of the mean correlation, estimated from bootstrapping the genes 100 times. The *p* values (red line) are based on a binomial test of the null hypothesis that equal numbers of genes have positive and negative correlations. (C) Rank correlation between the elongation *s*peed (measured at the ribosome A site) and experimentally determined *m*RNA folding strength (ρ_m-s_) at different offsets for individual genes. Genes are ordered according to their protein sequence conservation among orthologs. Rank correlation between ρ_m-s_ at offset +12 and the evolutionary conservation of the protein is −0.158 (*p*<10^−4^). In (B) and (C), only those genes that have ρ_m-s_ values for all offsets are shown. (D) Similar to (C), except that genes are ordered according to their expression levels in a rich medium. Rank correlation between ρ_m-s_ at offset +12 and the gene expression level is −0.320 (*p*<10^−47^). (E) Rank correlation (black dots) between the amino acid conservation of the codon being decoded and the mRNA folding strength at various offsets. Correlations are calculated for each gene and then averaged across 2,214 genes. Error bar indicates 95% confidence interval of mean correlation, estimated from bootstrapping the genes 100 times. The *p* values (red line) are based on a binomial test of the null hypothesis that equal numbers of genes have positive and negative correlations.

What could that information be? It was recently reported that mRNA folding strength and ribosome density are positively correlated [13], suggesting the possibility of mRNA secondary structures serving as elongation brakes. However, the previously shown correlation between mRNA folding and ribosome density was observed for 40-base stretches of mRNA, not specific enough for codon-level modulation of elongation speed. To test whether mRNA secondary structure modulates elongation speed and accuracy codon by codon, for each mRNA, we measured the rank correlation between the elongation speed of a codon at the ribosome A site and the local mRNA folding strength [30] of the nucleotide that has a predefined distance from the first nucleotide of the focal codon. Unless otherwise noted, the folding strength of a nucleotide site is defined as the probability that the nucleotide is paired in the mRNA secondary structure. The predefined distance is referred to as the offset, ranging from −20 to +20 bases in our analysis (Figure 6A). For each offset used, we then calculated the mean correlation from all genes. The strongest signal observed is a negative correlation at the offset of +12 bases (Figure 6B; see also Figure S4A and D), which is where mRNA enters the ribosome (Figure 6A) [12]. Our observation is not an artifact of read mapping ambiguity (Figure S4G and H; see Materials and Methods). It is further supported by *in vitro* RNA folding strengths quantified at a relatively high temperature (55°C) (Figure S4I; see Materials and Methods) [31], computationally predicted ribosome-bound RNA folding strengths (Figure S4J; see Materials and Methods), and *in vivo* measurements of RNA folding strengths [32] (Figure S4K; see Materials and Methods). These results strongly suggest that the elongation speed of a codon is influenced by the folding strength of its downstream nucleotide at the entrance of the ribosome, consistent with recent findings from *in vitro* single-molecule studies of translation [33],[34]. We found that the above correlation is stronger for genes with more conserved protein sequences (Figure 6C; see also Figure S4B and E) or higher expressions (Figure 6D; see also Figure S4C and F), where the demand for translational accuracy is greater (although this could also be due to better data quality for highly expressed genes). The impact of mRNA secondary structure appears to be independent from that of positively charged residues in the nascent peptide (Figure S4L), another determinant of the elongation speed [13],[14],[35]. To estimate the effect size of mRNA folding at offset +12 on elongation speed, we calculated, within each gene, the ratio in average ribosome density between codons with high and low RNA folding strengths at offset +12 (see Materials and Methods). In the 100 genes with the highest mRNA concentrations, the median of this ratio is at least ∼1.78 (Figure S4N), corresponding to a ∼40% reduction in elongation speed when a weakly folded nucleotide at offset +12 is changed to strongly folded. This effect is comparable in magnitude to the effect of positively charged residues on elongation speed [14] and is stronger than other proposed elongation decelerating mechanisms (see Materials and Methods) [36],[37], but none of these factors appear to modulate the speed–accuracy tradeoff (see below).

If local mRNA folding is the mechanism by which the tradeoff between translational accuracy and elongation speed is modulated, the amino acid conservation of a codon, reflecting the demand for translational accuracy, should be positively correlated with the mRNA folding strength at offset +12. Furthermore, a positive correlation at offset 0 is also expected, because if strong mRNA folding at a nucleotide position is important, mutations at the position will tend to be deleterious and purged by natural selection; consequently, the nucleotide and the codon containing the nucleotide are likely to be conserved evolutionarily [38]. Consistent with these predictions, the two highest peaks in the offset-correlation plot are found at offsets 0 and +12, respectively (Figure 6E). Because the folding strengths of neighboring nucleotides are highly similar (see Materials and Methods), the strong signal at offset −1 is likely due to its similarity with offset 0 in mRNA structure. Indeed, the signal is substantially reduced if a partial correlation at offset −1 is calculated after the control of mRNA folding strength at offset 0 (mean ρ = −0.010, binomial *p* = 3.54×10^−3^).

We found mRNA secondary structures to be the dominant mechanism optimizing the speed–accuracy tradeoff, because the within-gene rank correlations between the elongation speed of a codon and the conservation of its encoded amino acid no longer differ from the random expectations after the control of the mRNA folding strength at offset +12 (Figure S4J). We estimated that a single mutation that increases the mRNA folding strength of one nucleotide has a fitness effect ranging from 1.5×10^−6^ to 7×10^−3^ (i.e., 0.00015% to 0.7% fitter), depending on the mRNA concentration (see Materials and Methods). Because this number greatly exceeds the inverse of the effective population size (∼10^7^) of yeast [39], such mRNA folding-altering mutations can be targeted by natural selection.

### Experimental Validation

**Figure 7:**
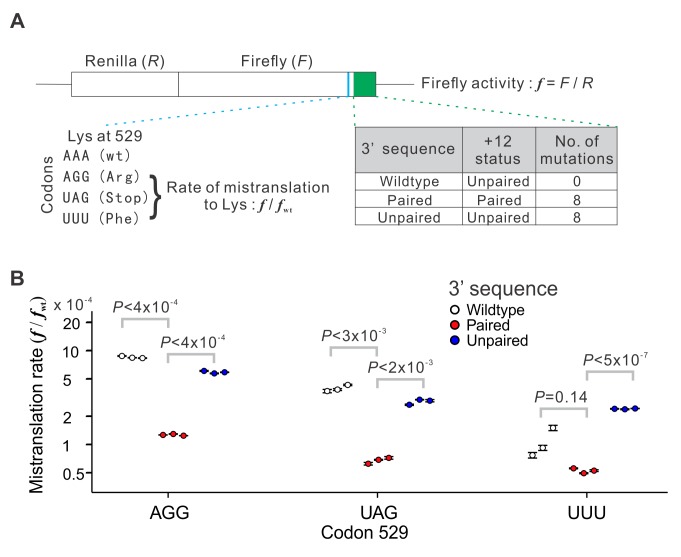
Dual luciferase assay demonstrating the impact of the pairing status of the offset +12 nucleotide in mRNA on mistranslation rate. (A) Experimental design. Concentration-independent firefly activity (*f*) is measured by the ratio between the firefly (*F*) and Renilla (*R*) signals. The firefly lysine codon AAA at position 529 (marked in blue in the fusion gene) is replaced with AGG, UAG, and UUU in three mutants, respectively. Because only protein molecules with lysine at position 529 would display luciferase activity, the rate of mistranslation to lysine at position 529 can be estimated by *f*/*f*_wt_, where *f*_wt_ is the *f* value for the wild-type (wt) (i.e., AAA at codon 529). Three versions of the 3′ sequence (region depicted in green in the fusion gene) are respectively used for the white, red, and blue dots in panel (B). (B) The rate of mistranslation to lysine at codon 529 (*f*/*f*_wt_) is influenced by the pairing status at the +12 nucleotide. Each genotype was measured in three biological replicates, depicted by three dots. Each dot represents the mean value from four technical repeats of each biological replicate, and the error bar shows the associated standard error. The *p* values are from *t* tests based on the three biological replicates. Note that for UUU, each white dot is higher than each red dot, which has a probability of 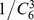 = 0.05 under the null hypothesis of no difference between white and red dots.

To experimentally verify the impact of mRNA folding at offset +12 on translational accuracy, we used a yeast dual luciferase reporter system [40] to quantify the mistranslation rate (Figure 7A). This system contains a chromosome-bound transgene that produces two luciferases, Renilla and firefly, in a fusion protein, allowing the measurement of concentration-independent firefly activity by the ratio between the observed firefly (*F*) and Renilla (*R*) activities. We used three mutants of the firefly segment of the fusion gene, in which codon AAA (Lys) at position 529 was replaced with TTT (Phe), TAG (Stop), and AGG (Arg), respectively. In these mutants, only proteins with mistranslation to Lys at position 529 can display a firefly activity, because no other side chain interacts with the luciferase substrate as the Lys side chain does [40],[41]. Consequently, the *F*/*R* ratio of the mutant, relative to that of the wild-type (i.e., AAA at position 529), measures the translational error rate [40]. Our model predicts that the mistranslation rate at codon 529 is influenced by the mRNA folding strength at offset +12. We computationally predicted the folding strength at offset +12 using its 3′ sequence, totaling 57 nucleotides from the +12 site to the stop codon. In the wild-type firefly mRNA, the +12 site relative to codon 529 is unpaired, and the only possible synonymous mutation at this site does not render it paired. To create a “paired” version in which the +12 site is paired, we swapped the synonymous codons in the 3′ sequence such that the protein sequence, nucleotide composition, and codon usage are all unaltered. The “paired” version we chose has eight synonymous differences and a greater mRNA folding strength, compared with the wild-type (see Materials and Methods). We similarly swapped the synonymous codons in the 3′ sequence to create an “unpaired” version as a negative control, which also contains eight synonymous differences from the wild-type, but with an unpaired +12 site and a relatively low mRNA folding strength (see Materials and Methods). We quantified the firefly and Renilla activities for all 12 constructs (one wild-type and three mutants at position 529 combined with three versions of the 3′ sequence) and calculated their associated mistranslation rates. When the wild-type 3′ sequence was used, mistranslation rates at position 529 measured in our experiments (Figure 7B) resemble those previously reported [40]. Note that although Kramer et al. originally thought that codon TTT cannot be mistranslated to Lys and thus regarded the TTT mutant as a negative control, their experimental data showed otherwise [40]. Specifically, they reported that the firefly/Renilla ratio is significantly higher for the TTT mutant than several other mutants they used [40]; the only viable explanation is that the mistranslation rate is nonzero and is higher for TTT than for these other mutants. Our result for the TTT mutant (Figure 7B) is consistent with theirs [40].

In support of our hypothesis, for each of the three mutants of codon 529, pairing at the +12 site in mRNA reduces the mistranslation rate by at least 50%, compared with their respective wild-type versions where the +12 site is unpaired (Figure 7B). Furthermore, unpairing the +12 site in our negative control more or less brought back the mistranslation rate to the original level (Figure 7B). These results confirm the effect of mRNA secondary structure, especially the pairing status at offset +12, on translational accuracy, and demonstrate that the effect size can be substantial.

## Discussion

In this study, we demonstrated through mathematical modeling and empirical genomic data analysis that the conflict between translational accuracy and speed is mitigated by sacrificing the speed at codons requiring high accuracy while compromising the accuracy for speed at other codons. By correlating between elongation speed and mRNA folding strength, we discovered that the adaptive tuning of translational speed and accuracy is achieved by differential mRNA folding at offset +12, which serves as a brake to control the elongation speed. Finally, we experimentally validated the impact of mRNA folding at offset +12 on translational accuracy by manipulating the DNA sequence of a transgene in yeast. Taken together, these results provide a unified model of codon-by-codon modulation of translational speed and accuracy, substantially improving our understanding of the translational process and its regulation.

Strong mRNA folding at offset +12 is expected to delay translocation during elongation [33],[34], but under the current model [5],[42],[43], eukaryotic translational fidelity is ensured during codon selection at the ribosome A site. Although we experimentally demonstrated the impact of downstream mRNA folding on translational fidelity, the underlying molecular details are unclear. One possibility is that mRNA folding at offset +12 induces a ribosomal conformational change that alters the kinetics of codon selection (e.g., the tRNA acceptance/rejection rate ratio), which could affect the elongation speed and accuracy (Figure S1A; see also Materials and Methods). Solving the structure of the translating ribosome–tRNA–mRNA complex [44],[45] with different mRNA folding strengths at offset +12 may offer direct evidence for this model. In addition, tracking the GTP hydrolysis rate during translation [7] and single-molecule studies [33],[34] may also help understand the molecule mechanism involved. Most of our genomic analysis (except Figure S4J–K) is based on *in vitro* mRNA folding strengths [30], which are only proxies of *in vivo* strengths. Thus, the actual impact of mRNA folding is expected to exceed what Figure 6 reveals. Furthermore, our findings imply that mRNA folding *in vivo* is environment-dependent [46], such that the same mRNA would fold somewhat differently to allow environment-specific tuning of elongation speed. RNA-binding proteins potentially play an important role in environment-specific mRNA folding; future studies should aim to identify these proteins and understand their environment-specific regulations. Interestingly, Figure 6B also suggests a positive effect of mRNA folding at offset −13 on elongation speed (*p*<10^−21^), although this effect is an order of magnitude weaker than the negative effect of mRNA folding at offset +12 (on the basis of fraction of variance explained). The positive effect at offset −13 requires further validation and its biological consequences are currently unknown. Another documented effect of mRNA folding on translational accuracy is pseudoknot triggered frame-shifting [47].

Although our experiment directly measured translational accuracy, it was done at only one locus. Systematic estimation of translational accuracy with reasonable sensitivity is nontrivial, because systems like the one used here are not scalable to the entire genome. However, with rapid improvements of proteomic techniques such as high-coverage mass spectrometry [48], it may be possible to measure translational errors for a large fraction of the proteome in the near future, and our results should be verified at a large scale at that time.

CUB was widely thought to affect elongation speed. But our analysis, along with several recent reports [3],[15],[25], argues against the role of CUB in regulating the elongation speed in wild-type cells. The interaction between positively charged amino acids in the nascent peptide and the negatively charged ribosome exit tunnel [14] and mRNA folding at the +12 site appear to be independent factors impacting the elongation speed (Figure S4L). However, the occurrence of positively charged amino acids is presumably determined largely by the structure and function of a protein and thus cannot be frequently deployed for regulating the elongation speed. By contrast, the degeneracy of the genetic code allows a certain degree of flexibility in using mRNA secondary structures for regulating the elongation speed. Consistent with this logic, we found mRNA secondary structures to be the dominant mechanism optimizing the speed–accuracy tradeoff (Figure S4M).

Our results contrast the common belief of faster elongation of more abundant mRNAs [49]. Rather, due to the high demand for translational accuracy of abundant proteins and the tradeoff between accuracy and speed, natural selection has resulted in slower translational elongation of more abundant mRNAs. Our findings suggest that the enigmatic positive correlation between gene expression level and mRNA folding strength [38],[50] at least partially results from selection for slower translational elongation of more abundant mRNAs to minimize mistranslation. Strong pressure for translational fidelity at a codon maintains an mRNA folding requirement at its +12 position and leads this position to evolutionary conservation. Because the demand for translational accuracy increases with expression level, the above mechanism brings an additional constraint to the evolution of highly expressed proteins [8],[10],[11],[38].

We previously demonstrated that, to minimize ribosome sequestration, transcriptomic synonymous codon usage should be proportional to the concentrations of their cognate tRNAs such that the cellular demand and supply of tRNAs are balanced [3]. This model is unaffected by the present finding of variable elongation speeds within and between genes, because the time needed for recycling a tRNA is much longer than the codon selection time (see Materials and Methods); consequently, the supply of tRNA is dictated by the tRNA recycling time (Figure S3E). Nonetheless, codon choice at a position may be constrained by the accuracy requirement at its −12 position, adding yet another layer of complexity to CUB.

Similar to protein translation, many biological processes face a tradeoff between efficiency and accuracy. A well-known example is in genome replication. It was observed that RNA viruses employ two unique strategies for genome replication, namely the “geometric replication” mode and the “stamping machine” mode. In the geometric replication mode, each RNA strand serves as a template for the synthesis of complementary strands with the same efficiency, whereas in the stamping machine mode, a strand is reiteratively used as a template to synthesize multiple copies of the complementary. Compared with the latter, the former mode is efficient but error-prone [51]. It was reported that the turnip mosaic virus (+) and (−) RNA amplification occurs through a mixed strategy, with 93% of genomes via stamping machine and 7% resulting from geometric replication [51]. Another documented tradeoff between efficiency and accuracy occurs in co-translational protein folding. Computational models predict that slowing the translation can increase the accuracy of co-translational folding [52]. Experimentally, disruption of a cluster of unpreferred codons has been shown to affect protein folding [53]. Given that unpreferred codons are not translated slower than preferred codons [3],[15],[25], the experimental result may be due to a change in elongation speed caused by an alteration in mRNA secondary structure. Although selection for co-translational folding accuracy can be an additional reason for a reduced speed of elongation, we were not able to detect a correlation between the elongation speed and protein structural features. This is probably because of the complexity of co-translational protein folding that makes it difficult to have a universal offset between structurally important sites and ribosome stalling codons.

In a more general context, although it is impossible to simultaneously maximize both efficiency and accuracy, it is often possible, at least in principle, to find optimal solutions in which the competing demands are mutually compromised in such a fashion that the resulting fitness is maximized [54]. Our finding of the exquisitely modulated translational speed and accuracy at the codon level is a strong testimony that such conflicts can and have been alleviated through mutation and selection. Because the efficiency–accuracy conflict is a form of antagonistic pleiotropy (AP), our results echo the recent report that AP is often resolvable, at least in part, when there is sufficient selection [55]. The molecular mechanisms of AP resolution, however, prove to be diverse [55].

## Materials and Methods

### Overview of Statistical Analyses

All statistical analyses were carried out in R [56]. We included as many data points as possible in each analysis unless otherwise stated. Consequently, the number of genes used in one test may differ from that in another test, depending on the data available for each test.

### Yeast Genomic Data

Protein and mRNA sequences of *Saccharomyces cerevisiae* were retrieved from the *Saccharomyces* Genome Database [57]. Protein sequences of five other post-WGD (whole-genome duplication) fungal species (*S. paradoxus*, *S. mikatae*, *S. bayanus*, *Candida glabrata*, and *S. castellii*) and gene orthology information among the six species were downloaded from the Fungal Orthogroups Repository [58]. Only one-to-one orthologs in all six species were used in our analysis. We aligned orthologous protein sequences by ClustalW [59]; alignment columns with gaps in any sequence were removed. The resulting alignments were used to estimate the mean substitution rate of all residues in each protein by PAML [60] and site-specific substitution rates of each protein by GAMMA [61]. The evolutionary conservation of a residue is the inverse of its substitution rate, whereas the mean evolutionary conservation of a protein is the inverse of its mean substitution rate. The *in vitro* experimental data from parallel analysis of RNA structure (PARS) [30] were used as the measurement of yeast mRNA folding strength. The PARS score of a nucleotide is the ratio between the relative probability that it is paired and the relative probability that it is unpaired. It has been reported that the first 30 to 50 codons of an mRNA form a “ramp” that reduces ribosomal traffic jam in downstream translation [19] and/or are less folded to allow translational initiation [4],[20],[62]. To avoid the interferences of these signals, we removed the first 50 codons from each coding sequence in our analysis. Stop codons were also removed because their ribosome densities reflect the time needed for translational termination.

### Mouse Genomic and Comparative Genomic Data

We downloaded one-to-one orthologous gene pairs between mouse (*Mus musculus*) and rat (*Rattus norvegicus*), as well as their protein sequences, from EnsEMBL (v69) [63]. Each pair of orthologous protein sequences were aligned by ClustalW [59]. After removing alignment gaps, we used PAML [60] to estimate the mean substitution rate of all residues of each protein.

### Yeast Ribosome Profiling Data

The high-throughput sequencing reads generated by the nucleotide-resolution ribosome profiling and mRNA-seq were downloaded from NCBI Gene Expression Omnibus (GEO) [64], under the accession number GSE13750 [12] and GSE34082 [22]. See Text S1 for details of the analysis of the ribosome profiling data.

### Translational Initiation Rates and Elongation Speeds of Yeast mRNAs

See Text S2 for details.

### Odds Ratios and the MH Test

See Text S3 for definitions and calculations of odds ratios (*OR*_1_, *OR*_2_, and *OR*_3_). The function “mantelhaen.test” provided by package “stats” in R was used to perform the MH test, which is also known as the Cochran–Mantel–Haenszel chi-squared test.

### Competing Demands for Translational Accuracy and Elongation Speed

See Text S4 for a detailed model of this problem.

### Ribosome Run-Off Data from Mouse Embryonic Stem Cells

The data were previously published [25]. See Text S5 for details of our analysis of this dataset.

### Selective Strength on Point Mutations Affecting the mRNA Secondary Structure

See Text S6 for detailed estimation.

### Amino Acid Charge and Elongation Speed

See Text S7 for detailed analysis.

### Quantifying Mistranslation Rates by Dual Luciferase Assays

The experiment generally followed an earlier study [40]. See Text S8 for details.

### Differential Elongation Speeds and Balanced Codon Usage

See Text S9 for details.

### Estimating Similarities in mRNA Folding Strength Between Neighboring Nucleotides

See Text S10 for details.

### A Simplified Model Explaining the Efficiency–Accuracy Tradeoff in Translation

See Text S11 for details.

### Robustness of the Effect of mRNA Folding Strength at Offset +12 on Elongation Speed

See Text S12 for details.

### Estimating Effect Sizes Related to mRNA Folding, Elongation Speed, and Evolutionary Conservation

See Text S13 for details.

## Supporting Information

Figure S1 Tradeoff between elongation speed and accuracy. (A) A cartoon illustrating the tradeoff between elongation speed and accuracy mediated by the tRNA acceptance/rejection rate ratio. For a given codon, the cognate tRNA molecules in the cell are shown by circles and the noncognate tRNAs are shown by rectangles. We use white and black symbols to represent the tRNAs that are accepted and rejected by the ribosome, respectively. That is, the acceptance to rejection rate ratio is the number of white symbols divided by the number of black symbols. According to ribosomal kinetics, the ratio between the acceptance/rejection rate ratio of the cognate tRNA and that of the noncognate tRNA is a constant (see Text S11) [7]. That is, (Wc∶Bc)/(Wn∶Bn) is a constant, where Wc, Bc, Wn, and Bn are the numbers of white cognate, black cognate, white noncognate, and black noncognate symbols, respectively. Note that (Wc∶Bc)/(Wn∶Bn) is equivalent to *d* in Eqs. [12], [13], and [14] (see Text S4). The existence of an efficiency–accuracy tradeoff can be proven mathematically (see Text S4). As an example, we arbitrarily assign (Wc∶Bc)/(Wn∶Bn) = 3 and the probability for the ribosome to encounter a cognate tRNA to be 50%. The top row of the figure shows relatively low acceptance/rejection rate ratios, whereas the bottom row shows relatively higher acceptance/rejection rate ratios. It is obvious that when the acceptance/rejection rate ratios are low, the elongation speed is low, because the ribosome needs to wait longer to have a tRNA accepted. The speed can be calculated by the fraction of white symbols among all symbols. The accuracy is the faction of white circles among all white symbols. The types of symbols counted in each equation are indicated in the gray-shaded regions below the numbers. One can see that the top row has a higher accuracy than the bottom row. (B–C) These panels are the same as Figure 1A and B, except that the yeast strain used is A14201. (D–E) These panels are the same as Figure 1A and B, except that the yeast strain used is gb15. Spearman's rank correlation of the original unbinned data is shown in (B–E). (F) This panel is the same as Figure 1C, except that here the ribosome profiling and mRNA-seq data are from two replicated experiments under the same rich media [12]. The replicated data are treated as if they are from two different environments. The correlation observed in Figure 1C now vanishes, suggesting that the original observation in Figure 1C is not an artifact of our analytical pipeline. See also Text S2.
(PDF)

Figure S2 Within individual yeast genes, codons with higher demands for translational accuracy have slower elongations. (A–C) These panels are the same as Figure 4A–C, except that the yeast strain used is A14201. (D–F) These panels are the same as Figure 4A–C, except that the yeast strain used is gb15.
(PDF)

Figure S3 Among residues encoded by the same codon within the same gene, those with higher demands for translational accuracy have lower elongation speeds. (A–B) These panels are the same as Figure 5A–B, except that the yeast strain used is A14201. (C–D) These panels are the same as Figure 5A–B, except that the yeast strain used is gb15. (E) Differential elongation speeds hardly affect the supply of different tRNAs, because all tRNAs spend most of their times in recycling. The percentage of time spent in recycling and translation for an average tRNA was estimated previously [3]. The relative amount of translation time required for slow, fast, and average codons are estimated by expression-weighted averages of 1/*v* for the 5% most abundant, 5% least abundant, and all mRNAs, respectively. Here *v* is the harmonic mean elongation speed of a gene.
(PDF)

Figure S4 Differential mRNA folding is the primary mechanism modulating the tradeoff between elongation speed and translational accuracy. (A–C) These panels are the same as Figure 6B–D, except that the yeast strain used is A14201. (D–F) These panels are the same as Figure 6B–D, except that the yeast strain used is gb15. (G) This panel is the same as Figure 6B, except that only ribosome-protected fragments that end in G or C are considered. See Text S12 for details. (H) This panel is the same as Figure 6B, except that only reads (from either ribosome profiling or mRNA-seq) that are uniquely aligned to the yeast genome are used. See Text S12 for details. (I) This panel is the same as Figure 6B, except that RNase V1 digestion-based *in vitro* mRNA folding strengths measured at 55°C [31] instead of PARS (measured at room temperature) are used, such that only energetically stable base-pairings remain. See Text S12 for details. (J) This panel is the same as Figure 6B, except that computationally predicted ribosome-bound mRNA folding strengths instead of PARS are used. See Text S12 for details. (K) This panel is the same as Figure 6B, except that *in vivo* DMS-based measurements [32] instead of PARS are used as mRNA folding strengths. See Text S12 for details. (L) Impact of mRNA folding strength (PARS) on elongation speed is independent from that of positively charged amino acids. Rank correlation (black dots) between the elongation speed (measured at the ribosome A site) and mRNA folding strength at different offsets, after the removal of all codons at ribosome A site, for which there are at least five positively charged amino acids in its preceding 30 codons. The correlations are calculated for each gene and then averaged across 358 genes. Error bar indicates 95% confidence intervals, estimated from bootstrapping the genes 1,000 times. The *p* values (red line) are based on a binomial test of the null hypothesis that equal numbers of genes have positive and negative correlations. The ribosome profiling dataset of Ingolia et al. [12] was used. See Text S7 and Text S12 for details. (M) Cumulative probability distributions for the observed and randomly expected within-gene partial rank correlations between the evolutionary conservation of the encoded amino acid of a codon and its elongation speed, after the control of mRNA folding strength (PARS) at offset +12. KS test, Kolmogorov–Smirnov test of the equality of the two distributions. The ribosome profiling dataset of Ingolia et al. [12] was used. (N) The effect of mRNA folding strength at offset +12 on ribosome density. Within each gene, we calculated the ratio in average ribosome density between codons with high folding strengths at offset +12 and those with low folding strengths at offset +12. High (or low) folding strength is defined as the highest (lowest) 5%, 10%, 20%, 30%, 40%, or 50% (the x axis) of the PARS values within each gene. The median ratio (y axis) in the 100 genes with the highest expressions is showed. Error bars indicate 95% confidence intervals, estimated from bootstrapping the genes 1,000 times. See Text S13 for details.
(PDF)

Text S1 Yeast ribosome profiling data.
(DOC)

Text S2 Translational initiation rates and elongation speeds of yeast mRNAs.
(DOC)

Text S3 Odds ratios and MH test.
(DOC)

Text S4 Competing demands for translational accuracy and elongation speed.
(DOC)

Text S5 Ribosome run-off data from mouse embryonic stem cells.
(DOC)

Text S6 Selective strength on point mutations affecting mRNA secondary structures.
(DOC)

Text S7 Amino acid charge and elongation speed.
(DOC)

Text S8 Quantifying mistranslation rates by dual luciferase assays.
(DOC)

Text S9 Differential elongation speeds and balanced codon usage.
(DOC)

Text S10 Similarity in PARS between neighboring nucleotides.
(DOC)

Text S11 Explanation of the simplified model in Figure S1A.
(DOC)

Text S12 Robustness of the effect of mRNA folding at offset +12 on elongation speed.
(DOC)

Text S13 Comparing the effect sizes on elongation speed by various factors.
(DOC)

## Abbreviations

AP: antagonistic pleiotropy
CUB: codon usage bias
MH test: Mantel–Haenszel test
OR: odds ratio
PARS: parallel analysis of RNA structure
WGD: whole-genome duplication
