# Supplementary figures and images for: Codon-by-Codon Modulation of Translational Speed and Accuracy Via mRNA Folding
Source: PLoS Biol. 2014 Jul 22;12(7):e1001910. doi: 10.1371/journal.pbio.1001910 (PMC4106722; doi:10.1371/journal.pbio.1001910)

**A**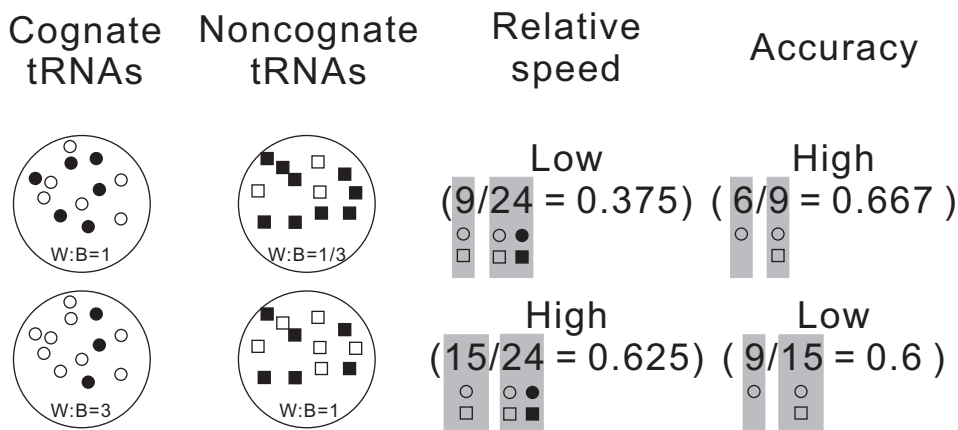**B**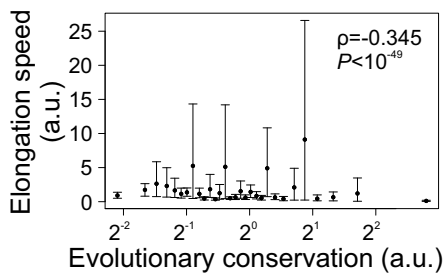**D**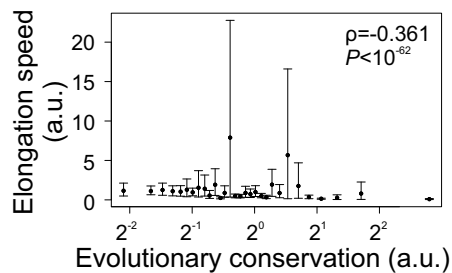**C**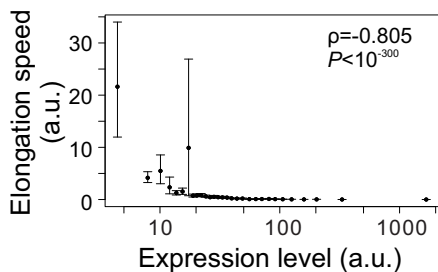**E**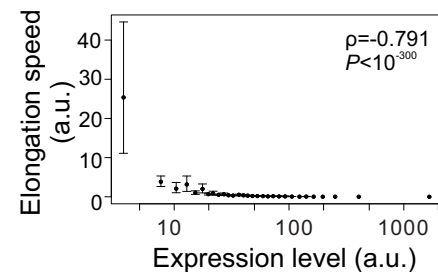**F**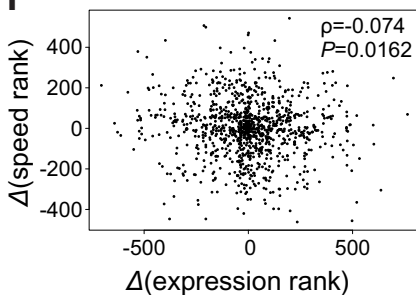

Figure S1

Supplement: Figure S1 — Tradeoff between elongation speed and accuracy. (A) A cartoon illustrating the tradeoff between elongation speed and accuracy mediated by the tRNA acceptance/rejection rate ratio. For a given codon, the cognate tRNA molecules in the cell are shown by circles and the noncognate tRNAs are shown by rectangles. We use white and black symbols to represent the tRNAs that are accepted and rejected by the ribosome, respectively. That is, the acceptance to rejection rate ratio is the number of white symbols divided by the number of black symbols. According to ribosomal kinetics, the ratio between the acceptance/rejection rate ratio of the cognate tRNA and that of the noncognate tRNA is a constant (see Text S11) [7]. That is, (Wc∶Bc)/(Wn∶Bn) is a constant, where Wc, Bc, Wn, and Bn are the numbers of white cognate, black cognate, white noncognate, and black noncognate symbols, respectively. Note that (Wc∶Bc)/(Wn∶Bn) is equivalent to d in Eqs. [12], [13], and [14] (see Text S4). The existence of an efficiency–accuracy tradeoff can be proven mathematically (see Text S4). As an example, we arbitrarily assign (Wc∶Bc)/(Wn∶Bn) = 3 and the probability for the ribosome to encounter a cognate tRNA to be 50%. The top row of the figure shows relatively low acceptance/rejection rate ratios, whereas the bottom row shows relatively higher acceptance/rejection rate ratios. It is obvious that when the acceptance/rejection rate ratios are low, the elongation speed is low, because the ribosome needs to wait longer to have a tRNA accepted. The speed can be calculated by the fraction of white symbols among all symbols. The accuracy is the faction of white circles among all white symbols. The types of symbols counted in each equation are indicated in the gray-shaded regions below the numbers. One can see that the top row has a higher accuracy than the bottom row. (B–C) These panels are the same as Figure 1A and B, except that the yeast strain used is A14201. (D–E) These panels are the same as Figu [file pbio.1001910.s001.pdf]

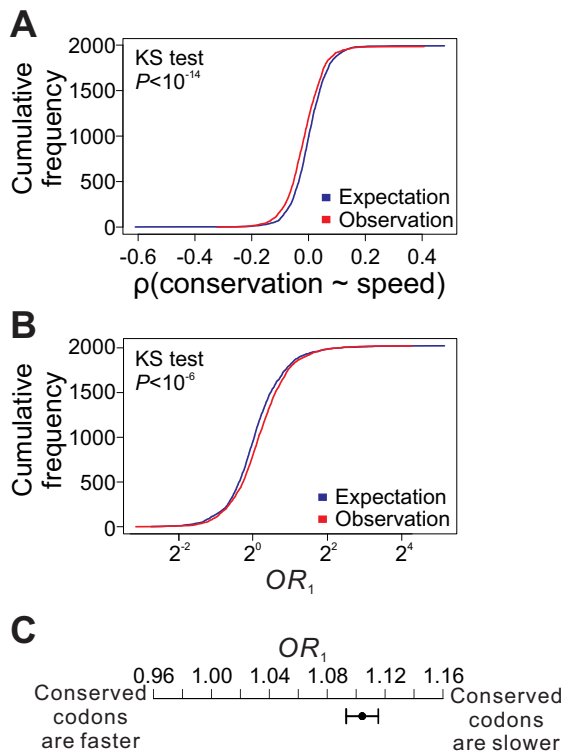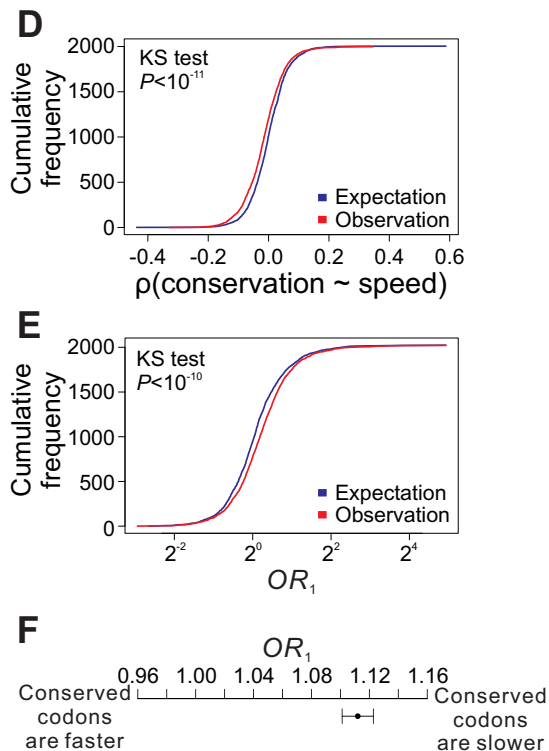

**Figure S2**

Supplement: Figure S2 — Within individual yeast genes, codons with higher demands for translational accuracy have slower elongations. (A–C) These panels are the same as Figure 4A–C, except that the yeast strain used is A14201. (D–F) These panels are the same as Figure 4A–C, except that the yeast strain used is gb15. (PDF) [file pbio.1001910.s002.pdf]

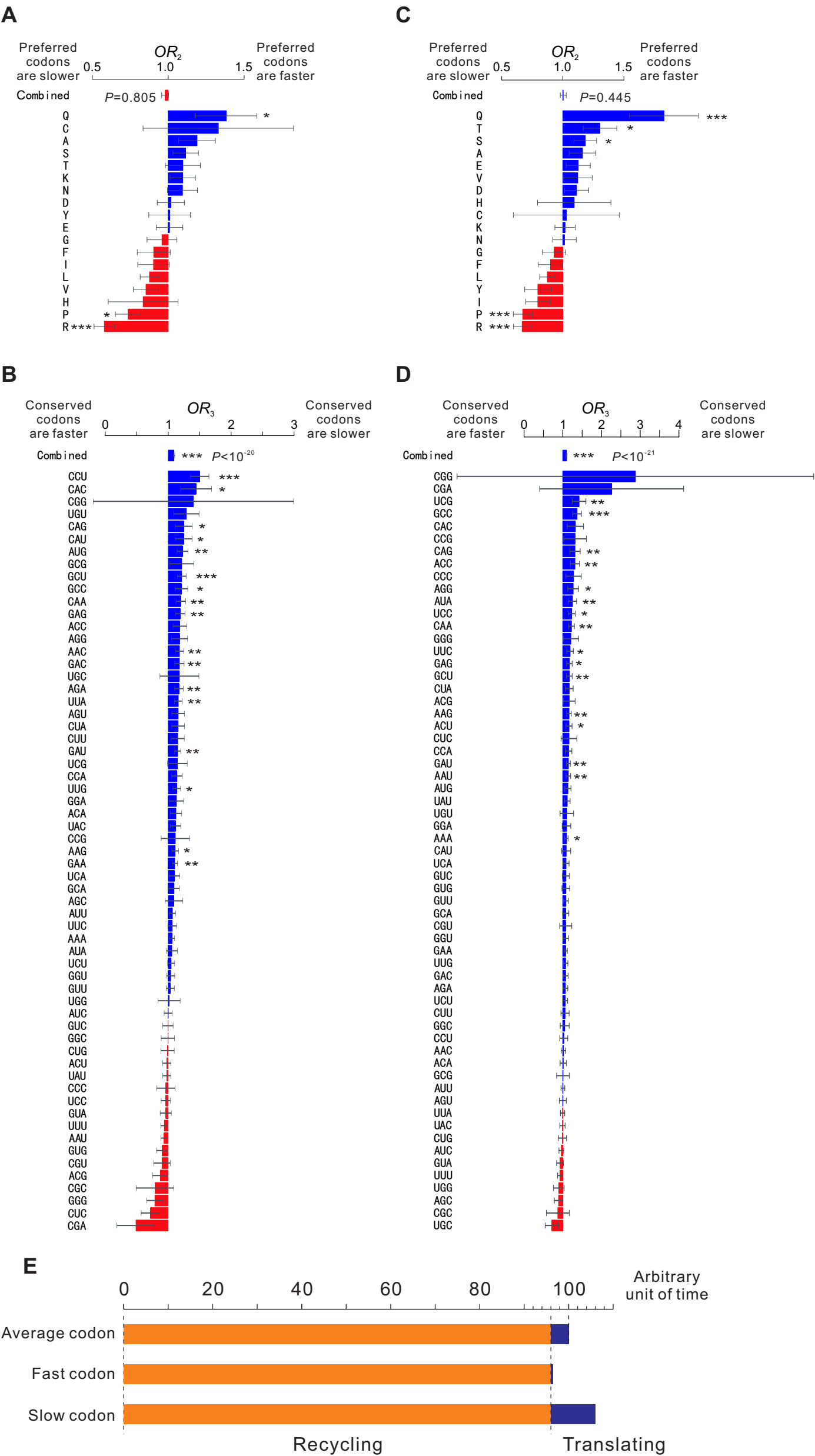

Figure S3

Supplement: Figure S3 — Among residues encoded by the same codon within the same gene, those with higher demands for translational accuracy have lower elongation speeds. (A–B) These panels are the same as Figure 5A–B, except that the yeast strain used is A14201. (C–D) These panels are the same as Figure 5A–B, except that the yeast strain used is gb15. (E) Differential elongation speeds hardly affect the supply of different tRNAs, because all tRNAs spend most of their times in recycling. The percentage of time spent in recycling and translation for an average tRNA was estimated previously [3]. The relative amount of translation time required for slow, fast, and average codons are estimated by expression-weighted averages of 1/v for the 5% most abundant, 5% least abundant, and all mRNAs, respectively. Here v is the harmonic mean elongation speed of a gene. (PDF) [file pbio.1001910.s003.pdf]

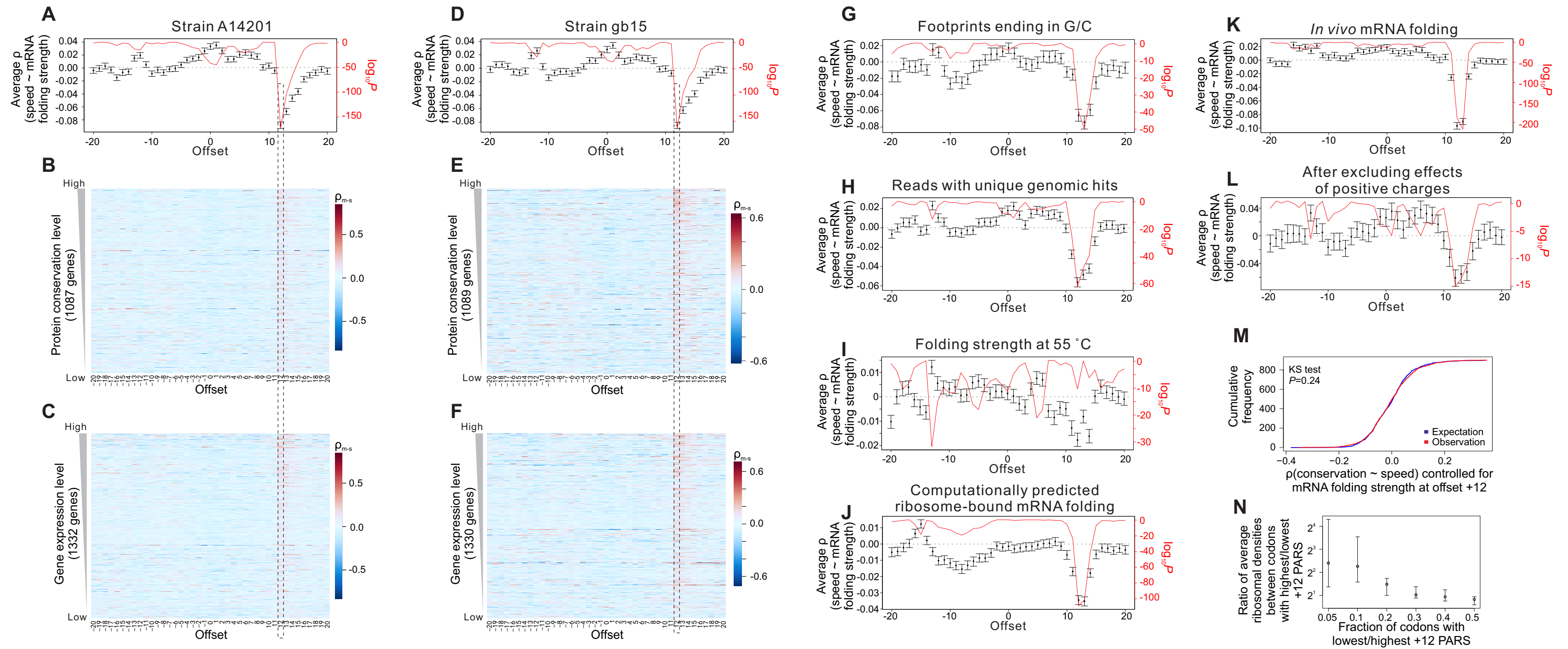

Figure S4

Supplement: Figure S4 — Differential mRNA folding is the primary mechanism modulating the tradeoff between elongation speed and translational accuracy. (A–C) These panels are the same as Figure 6B–D, except that the yeast strain used is A14201. (D–F) These panels are the same as Figure 6B–D, except that the yeast strain used is gb15. (G) This panel is the same as Figure 6B, except that only ribosome-protected fragments that end in G or C are considered. See Text S12 for details. (H) This panel is the same as Figure 6B, except that only reads (from either ribosome profiling or mRNA-seq) that are uniquely aligned to the yeast genome are used. See Text S12 for details. (I) This panel is the same as Figure 6B, except that RNase V1 digestion-based in vitro mRNA folding strengths measured at 55°C [31] instead of PARS (measured at room temperature) are used, such that only energetically stable base-pairings remain. See Text S12 for details. (J) This panel is the same as Figure 6B, except that computationally predicted ribosome-bound mRNA folding strengths instead of PARS are used. See Text S12 for details. (K) This panel is the same as Figure 6B, except that in vivo DMS-based measurements [32] instead of PARS are used as mRNA folding strengths. See Text S12 for details. (L) Impact of mRNA folding strength (PARS) on elongation speed is independent from that of positively charged amino acids. Rank correlation (black dots) between the elongation speed (measured at the ribosome A site) and mRNA folding strength at different offsets, after the removal of all codons at ribosome A site, for which there are at least five positively charged amino acids in its preceding 30 codons. The correlations are calculated for each gene and then averaged across 358 genes. Error bar indicates 95% confidence intervals, estimated from bootstrapping the genes 1,000 times. The p values (red line) are based on a binomial test of the null hypothesis that equal numbers of genes have positive and negative correlations. The ri [file pbio.1001910.s004.pdf]
