# Supplementary material for: Codon-by-Codon Modulation of Translational Speed and Accuracy Via mRNA Folding
Source: PLoS Biol. 2014 Jul 22;12(7):e1001910. doi: 10.1371/journal.pbio.1001910 (PMC4106722; doi:10.1371/journal.pbio.1001910)
Supplement: Text S1 — Yeast ribosome profiling data. (DOC) [file pbio.1001910.s005.doc]

**Text S1. Yeast ribosome profiling data**

In ribosome profiling, cycloheximide is used to stop translational elongation . Cycloheximide has been observed to allow the ribosome to translocate one codon before it stalls in the one translocation step immediately after translational initiation . The mechanism behind this phenomenon is that cycloheximide requires an E-site-bound deacylated tRNA for its activity . Given this mechanism, translocation must be stopped right after the cycloheximine treatment during elongation.

The high-throughput sequencing reads generated by the nucleotide-resolution ribosome profiling and mRNA-seq experiments for *S. cerevisae* strain BY4741 were downloaded from NCBI Gene Expression Omnibus (GEO) , under the accession number GSE13750 . All reads were previously mapped by Ingolia et al. to the yeast reference genome, allowing up to 3 mismatches. In our analysis, unless otherwise noted, reads from two replicated experiments were pooled together, and only mappings aligned to the genome with exactly 28 nucleotides, reported to be the number of nucleotides protected by a single eukaryotic ribosome, were used. The codon being decoded by the ribosome is the 16th-18th nucleotides of each 28-nucleotide read . If the 16th-18th nucleotides were out of the reading frame, the read was discarded. To deal with ambiguous mapping, we first considered only reads uniquely mapped to the yeast genome and calculated unique-hit read counts for each codon across the genome. Then, each read with multiple equally good matches was counted toward all potential targets in proportion to the unique-hit read counts around (i.e., 10 nucleotides before and after) these targets. Sequencing reads from mRNA-seq were processed in the same fashion as the ribosome profiling reads. Again, only 28-nucleotide reads with the correct reading frame were used. To control for sequencing bias, we followed a previous analysis to calculate the relative ribosome density at the *j*th codon of the *i*th mRNA by *d*_ij_ = *A*_ij_ / (*B*_ij_ + 1), where *A*_ij_ and *B*_ij_ are the read counts from ribosome profiling and mRNA-seq data for the codon, respectively. Use of this formula controls sequencing biases, because the biases in ribosome profiling and mRNA-seq are canceled out. The pseudocount of 1 was added to avoid dividing *A*_ij_ by 0, and using different pseudocounts yielded qualitatively similar results. For instance, when the pseudocount was set at 10^-4^, which is smaller than the minimal average *B*_ij_ for genes with at least one mRNA-seq read, the average elongation speed of a gene is still negatively correlated with both the average evolutionary conservation of its protein sequence (ρ = -0.340, *P* < 10^-50^) and its mRNA concentration (ρ = -0.644, *P*<10^-261^). Further, the change in the rank of gene expression between the two environments examined and the corresponding change in the rank of elongation speed remains negatively correlated (ρ = -0.321, *P* < 10^-40^). The effect of using a pseudocount is expected to be the same across all sites within a gene. Hence, the pseudocount should not affect any within-gene comparison. The two additional ribosome profiling datasets were from *S. cerevisae* strains A14201 and gb15, both with the SK1 background . The high-throughput sequencing reads generated from ribosome profiling and mRNA-seq experiments were also downloaded from GEO , under the accession number GSE34082 . They were then mapped to the yeast reference genome by bowtie2 in “--sensitive-local” mode. Only mappings with exactly 28-nt alignment and 0 mismatch to the genome were used. Downstream analyses to assess ribosome density were carried out in the same way as described above for the data from strain BY4741. Thus, results from the three strains are directly comparable.

The only other published yeast ribosome profiling dataset cannot be used for our study, because 28 to 32-nt ribosome footprints were retrieved for sequencing in the experiment . Consequently, the codon at the ribosome A site cannot be unambiguously determined.

**References**

1. Ingolia NT, Ghaemmaghami S, Newman JR, Weissman JS (2009) Genome-wide analysis in vivo of translation with nucleotide resolution using ribosome profiling. Science 324: 218-223.

2. Schneider-Poetsch T, Ju J, Eyler DE, Dang Y, Bhat S, et al. (2010) Inhibition of eukaryotic translation elongation by cycloheximide and lactimidomycin. Nat Chem Biol 6: 209-217.

3. Barrett T, Wilhite SE, Ledoux P, Evangelista C, Kim IF, et al. (2013) NCBI GEO: archive for functional genomics data sets--update. Nucleic Acids Res 41: D991-995.

4. Brar GA, Yassour M, Friedman N, Regev A, Ingolia NT, et al. (2012) High-resolution view of the yeast meiotic program revealed by ribosome profiling. Science 335: 552-557.

5. Langmead B, Salzberg SL (2012) Fast gapped-read alignment with Bowtie 2. Nat Methods 9: 357-359.

6. Gerashchenko MV, Lobanov AV, Gladyshev VN (2012) Genome-wide ribosome profiling reveals complex translational regulation in response to oxidative stress. Proc Natl Acad Sci U S A 109: 17394-17399.
