# Supplementary material for: Codon-by-Codon Modulation of Translational Speed and Accuracy Via mRNA Folding
Source: PLoS Biol. 2014 Jul 22;12(7):e1001910. doi: 10.1371/journal.pbio.1001910 (PMC4106722; doi:10.1371/journal.pbio.1001910)
Supplement: Text S2 — Translational initiation rates and elongation speeds of yeast mRNAs. (DOC) [file pbio.1001910.s006.doc]

**Text S2. Translational initiation rates and elongation speeds of yeast mRNAs**

Let the translational initiation rate of the *i*th mRNA be *I*_i_. The elongation speed for its codon *j* can be estimated by *e*_ij_ = *I*_i_/*d*_ij_ when ribosome drop-off is negligible . Under the same assumption, *I*_i_ equals the protein synthesis rate, which can be estimated by *I*_i_ = [*D*_i_+(ln2)/*T*]*P*_i_/*M*_i_, where *D*_i_ is the protein degradation rate, *P*_i_ is the protein concentration, *M*_i_ is the mRNA concentration, and *T* = 90 minutes is the generation time of yeast during exponential growth . We estimated *M*_i_ using the above mentioned mRNA-seq data and used immunodetection-based estimates of *P*_i_ and *D*_i_ . Combining the above two equations yields *e*_ij_ = [*D*_i_+(ln2)/90]*P*_i_/(*M*_i_*d*_ij_). Note that mRNA-seq data were used to estimate *M*_i_, which is a denominator in calculating *d*_ij_. Consequently, the estimation of *e*_ij_ is independent from *M*_i_. *I*_i_ would have been underestimated in the presence of non-negligible ribosome drop-off. But results described in the following two paragraphs show that our results are insensitive to *I*_i_ estimates. Furthermore, within-gene analysis, which does not depend on *I*_i_, supports the hypothesis that codons with higher demands for accuracy have slower elongations. Non-negligible ribosome drop-off would also lead to overestimation of the elongation speed at the 3’ end of a gene, relative to that at the 5’ end. But, because codons with high demands for accuracy are not expected to be enriched at one end relative to the other, the potential estimation bias of the elongation speed along a gene would not compromise our analysis, although it likely reduces the power of our analysis.

For comparison among genes, we calculated average elongation speed of each gene. We first calculated average ribosome density across all codons in the gene and then calculated the average elongation speed from the average ribosome density. This is equivalent to calculating the harmonic mean elongation speed of mRNA *i*, or *E*_i_, from estimates of *e*_ij_. We found that *E*_i_ is negatively correlated with the average evolutionary conservation (*C*_i_) of the protein sequence of gene *i* (Fig. 1A). Similarly, we observed a negative correlation between *E*_i_ and *M*_i_ (Fig. 1B). One caveat in the above analyses is that estimates of *D*_i_ may be unreliable when *P*_i_ is too high or too low, because immunodetection has a limited dynamic range . To exclude the possibility that the negative correlation between *E*_i_ and *C*_i_ and that between *E*_i_ and *M*_i_ are artifacts caused by unreliable estimates of *D*_i_, we removed genes whose protein or mRNA concentrations are within the top 10% or bottom 10% of all genes. But the two correlations remained highly significant (*ρ* = -0.259, *P* < 10^-16^ for the correlation between *E*_i_ and *C*_i_; *ρ* = -0.596, *P* < 10^-114^ for the correlation between *E*_i_ and *M*_i_).

On the basis of the *D*_i_ estimates, we found a negative correlation between mRNA concentration *M*_i_ and translational initiation rate *I*_i_ in yeast (ρ = -0.482, *P* < 10^-130^), consistent with the previously reported strategy of gene expression noise minimization in yeast . This negative correlation, however, does not fully account for the negative correlation between *E*_i_ and *C*_i_ (Fig. 1A) or that between *E*_i_ and *M*_i_ (Fig. 1B). That is, even using the assumption of equal translational initiation rates among genes when estimating *E*_i_, we found the correlation between *E*_i_ and *C*_i_ to remain negative (ρ = -0.451, *P*<10^-93^) and that between *E*_i_ and *M*_i_ to remain negative too (ρ = -0.571, *P*<10^-192^). Using yeast translational initiation rates recently estimated by Shah and colleagues , we confirmed the negative correlation between *E*_i_ and *C*_i_ (ρ = -0.312, *P*<10^-38^) and that between *E*_i_ and *M*_i_ (ρ = -0.504, *P*<10^-126^).

When examining the correlation between the change in the expression level of a gene across two environments (YPD and amino acid starvation media) and the change in its elongation speed, we used the same initiation rate estimated under YPD for the gene regardless of the environment. If the initiation rate of a gene decreases when its expression level increases, as was found across genes under YPD in the paragraph above, our analysis would have overestimated the initiation rates and elongation speeds under the amino acid starvation medium for those genes with higher expressions in this medium than in YPD, rendering our finding of the negative correlation in Fig. 1C conservative. Furthermore, under the assumption of equal translational initiation rates among genes and across environments, the correlation between the change in the rank of gene expression across the two environments and the change in the rank of elongation speed remains significantly negative (ρ = -0.313, *P*<10^-38^).

Because two biological replicates of ribosome profiling were conducted under YPD , we used a comparison between these replicates as a negative control of the between-environment comparison of Fig. 1C. Specifically, we separately calculated the elongation speed using ribosome profiling and mRNA-seq data from each replicate experiment and then calculated the correlation between the change in the rank of gene expression across the two replicates and the change in the rank of elongation speed. In contrast to the strong negative correlation observed when two actually different environments are compared, the correlation vanishes when two replicates under the same environment are compared (Spearman’s ρ = -0.075, *P* = 0.0162; Fig. S1F). Thus, the observation in Fig. 1C is genuine and is not caused by any systematic bias in our analytic pipeline.

**References**

1. Qian W, Yang JR, Pearson NM, Maclean C, Zhang J (2012) Balanced codon usage optimizes eukaryotic translational efficiency. PLoS Genet 8: e1002603.

2. Belle A, Tanay A, Bitincka L, Shamir R, O'Shea EK (2006) Quantification of protein half-lives in the budding yeast proteome. Proc Natl Acad Sci U S A 103: 13004-13009.

3. Ingolia NT, Ghaemmaghami S, Newman JR, Weissman JS (2009) Genome-wide analysis in vivo of translation with nucleotide resolution using ribosome profiling. Science 324: 218-223.

4. Ghaemmaghami S, Huh WK, Bower K, Howson RW, Belle A, et al. (2003) Global analysis of protein expression in yeast. Nature 425: 737-741.

5. Fraser HB, Hirsh AE, Giaever G, Kumm J, Eisen MB (2004) Noise minimization in eukaryotic gene expression. PLoS Biol 2: e137.

6. Shah P, Ding Y, Niemczyk M, Kudla G, Plotkin JB (2013) Rate-limiting steps in yeast protein translation. Cell 153: 1589-1601.
