# Supplementary material for: Codon-by-Codon Modulation of Translational Speed and Accuracy Via mRNA Folding
Source: PLoS Biol. 2014 Jul 22;12(7):e1001910. doi: 10.1371/journal.pbio.1001910 (PMC4106722; doi:10.1371/journal.pbio.1001910)
Supplement: Text S3 — Odds ratios and MH test. (DOC) [file pbio.1001910.s007.doc]

**Text S3. Odds ratios and Mantel-Haenszel test**

We defined and calculated three odds ratio in our analyses, namely *OR*_1_, *OR*_2_, and *OR*_3_. To calculate *OR*_1_, a 2×2 table was constructed for each gene by classifying each codon in the gene into one of four categories based on (i) whether its ribosome density is ≤ the median ribosome density within the gene and (ii) whether its corresponding amino acid is conserved across six post-WGD (whole-genome duplication) fungal species (*S. cerevisiae*, *S. paradoxus*, *S. mikatae*, *S. bayanus*, *Candida glabrata*, and *S. castellii*). Let the numbers of codons that fall in the four categories be: *a*_1_ (yes to both questions), *b*_1_ (yes to only question i), *c*_1_ (yes to only question ii), and *d*_1_ (no to both questions), respectively. *OR*_1_ = (*b*_1_*c*_1_)/(*a*_1_*d*_1_). Thus, *OR*_1_ > 1, if conserved residues within a gene tend to have slower translational elongations. In Fig. 4C, the Mantel-Haenszel (MH) procedure was used to combine *OR*_1_ from all genes.

To calculate *OR*_2_, in each gene, a 2×2 table was constructed for each of the 18 amino acids that are encoded by at least two different synonymous codons. All codons encoding each amino acid were classified into four categories based on (i) whether the ribosome density of a codon is higher than the median ribosome density of all codons of the amino acid within the gene, and (ii) whether this codon is a preferred codon. To determine whether a codon is preferred, we used the relative synonymous codon usage (RSCU), defined by the frequency of a codon relative to the average frequency of all of its synonymous codons in 200 highly expressed genes . Codons with RSCU > 1 are preferred and those with RSCU < 1 are unpreferred. Let the numbers of codons that fall in the four categories be: *a*_2_ (yes to both questions), *b*_2_ (yes to only question i), *c*_2_ (yes to only question ii), and *d*_2_ (no to both questions), respectively. *OR*_2_ = (*b*_2_*c*_2_)/(*a*_2_*d*_2_). Thus, *OR*_2_ > 1, if preferred codons of an amino acid tend to have lower ribosome densities (i.e., faster elongations) in a gene. In Fig. 5A, the Mantel-Haenszel (MH) procedure was first used to combine *OR*_2_ of a given amino acid from all genes (results for individual amino acids), and then used to combine *OR*_2_ of all amino acids from all genes (the “combined” result).

To calculate *OR*_3_, a 2×2 table was constructed for each of the 61 sense codons in each gene. For any particular one of the 61 sense codons, all occurrences of the codon in the gene were classified into four categories based on (i) whether the ribosome density at a codon occurrence is ≤ the median ribosomal density of all occurrences of the codon within the gene, and (ii) whether its encoded amino acid is conserved across the aforementioned six fungal species. Let the numbers of codon occurrences that fall in the four categories be: *a*_3_ (yes to both questions), *b*_3_ (yes to only question i), *c*_3_ (yes to only question ii), and *d*_3_ (no to both questions), respectively. *OR*_3_ = (*b*_3_*c*_3_)/(*a*_3_*d*_3_). Thus, *OR*_3_ > 1, if, for a given sense codon, those positions with conserved residues tend to have higher ribosome densities (i.e., slower elongations) in a gene. In Fig. 5B, the Mantel-Haenszel (MH) procedure was first used to combine *OR*_3_ of a given codon from all genes (results for individual codons), and was then used to combine *OR*_3_ of all codons from all genes (the “combined” result).

**References**

1. Qian W, Yang JR, Pearson NM, Maclean C, Zhang J (2012) Balanced codon usage optimizes eukaryotic translational efficiency. PLoS Genet 8: e1002603.
