# Supplementary material for: Codon-by-Codon Modulation of Translational Speed and Accuracy Via mRNA Folding
Source: PLoS Biol. 2014 Jul 22;12(7):e1001910. doi: 10.1371/journal.pbio.1001910 (PMC4106722; doi:10.1371/journal.pbio.1001910)
Supplement: Text S4 — Competing demands for translational accuracy and elongation speed. (DOC) [file pbio.1001910.s008.doc]

**Text S4. Competing demands for translational accuracy and elongation speed**

Compared with lowly expressed genes, highly expressed genes are subject to stronger demands for both translational accuracy and elongation speed, which are in conflict. To predict how gene expression level impacts translational accuracy and speed, we built a mathematical model to calculate the fitness effects associated with translational accuracy and elongation speed, respectively. Here the fitness of the wild-type is defined as 1, and the fitness of any mutant is computed relative to the wild-type.

Let us first consider the benefit of reducing ribosome sequestering by increasing the elongation speed. Imagine a wild-type yeast strain with genome *G* and total number of translating ribosomes *R*. Let us assume that the mean elongation speed of a gene (ν) is the same among all genes. A mutation in gene *g* increases the mean elongation speed of the gene by Δν. Each cellular generation requires the translation of the whole proteome for a new cell and the renewal of degraded proteins. If protein synthesis is the limiting factor in cell division, the generation time of the wild-type strain can be expressed by

$T_{\text{wt}}=\frac{D_{\text{aa}}T_{\text{wt}}+\sum_{i} E_{i}L_{i}P_{i}}{Rv}$, [1]

where *D*_aa_ is the number of amino acids in the degraded proteins per minute per cell, and *E*_i_, *L*_i_, and *P*_i_ are the number of mRNA molecules per cell, protein length, and number of proteins synthesized per mRNA molecule per generation for gene *i*, respectively. The two items on the top part of the right side of the equation represent the number of amino acids in the proteins degraded per generation and the number of amino acids in the proteome of the new cell created every generation, respectively. For the mutant strain, the genome could be divided into two parts, the mutated gene *g* with elongation speed ν+Δν, and the other genes with elongation speed *v*. Under the assumption that the protein product of gene *g* has an average half-life, the generation time of the mutant (*T*_mt_) is

$T_{\text{mt}}=\frac{\frac{E_{g}L_{g}P_{g}}{\sum_{i} E_{i}L_{i}P_{i}}D_{\text{aa}}T_{\text{mt}}+E_{g}L_{g}P_{g}}{R(v+\Delta v)}+\frac{\frac{\sum_{i\neq g} E_{i}L_{i}P_{i}}{\sum_{i} E_{i}L_{i}P_{i}}D_{\text{aa}}T_{\text{mt}}+\sum_{i\neq g} E_{i}L_{i}P_{i}}{Rv}$. [2]

During exponential growth, population size grows according to

$N_{\text{wt}}(t+1)=2^{1/T_{\text{wt}}}N_{\text{wt}}(t) \text{and} N_{\text{mt}}(t+1)=2^{1/T_{\text{mt}}}N_{\text{mt}}(t)$, [3]

where *N*_wt_(*t*) and *N*_wt_(*t*+1) are the population sizes of the wild-type strain at time *t* and *t*+1, respectively, and *N*_mt_(*t*) and *N*_mt_(*t*+1) are the population sizes of the mutant strain at time *t* and *t*+1, respectively. The mutant’s fitness advantage (*s_v_*) over the wild-type due to the relief of ribosome sequestration is

$s_{v}=\frac{N_{\text{mt}}(t+T_{\text{wt}})/N_{\text{mt}}(t)}{N_{\text{wt}}(t+T_{\text{wt}})/N_{\text{wt}}(t)}-1=2^{T_{\text{wt}}/T_{\text{mt}}-1}-1$. [4]

Based on the literature, the best estimates for the parameters in this model are mean *L* ≈ 400 codons, mean *P* ≈ 5,000 , *R* ≈ 200,000 , $\sum_{i} E_{i}$≈ 12,000 , *D*_aa_ ≈ 100,000*L*/60 per second , and baseline *v* = 20 codons per second . We plotted *s_v_* for various values of Δν and *E_g_* (Fig. 2A). As expected, a positive Δν results in a positive fitness advantage, and vice versa. Given Δ*ν*, the absolute value of *s_v_* is greater when $\Delta v$occurs to a highly expressed gene than to a lowly expressed gene. We also found that, given *E_g_*, the fitness advantage does not increase linearly with Δ*ν*, but shows a diminishing return, reflected by the increasing distances between the contour lines when Δ*ν* increases (Fig. 2A). This phenomenon is not unexpected, because as Δ*ν* in gene *g* increases, ribosomes spend a larger fraction of time on genes other than *g*, effectively reducing the benefit of the increased elongation speed in *g*.

Let us now consider the cost of translational error caused by increasing the elongation speed. It is reasonable to assume that the growth rate of the mutant is

$r_{\text{mt}}=r_{\text{wt}}-c_{1}M_{\text{mt-wt}}$, [5]

where *r*_wt_ is the growth rate of the wild-type strain, *c*_1_ is a constant, and *M*_mt-wt_ is the number of additional mistranslation-induced misfolded proteins produced per second in the mutant, compared with that in the wild-type . Let us assume that the translational error rate per residue is *a*_mt_ and *a*_wt_ in the mutant and wild-type strains, respectively. Then

$M_{\text{mt-wt}}=E_{g}P_{g}f_{t}\left[\left(1-a_{\text{wt}}\right)^{L_{g}}-\left(1-a_{\text{mt}}\right)^{L_{g}}\right]$, [6]

where *f*_t_ is the fraction of mistranslated proteins that are misfolded. The growths of the wild-type and mutant populations respectively follow

$N_{\text{wt}}(t+1)=e^{r_{\text{wt}}}N_{\text{wt}}(t) \text{and} N_{\text{mt}}(t+1)=e^{r_{\text{wt}}-c_{1}M_{\text{mt-wt}}}N_{\text{mt}}(t)$. [7]

Because $e^{r_{\text{wt}}T_{\text{wt}}}=2$, the fitness advantage of the mutant, relative to the wild-type, is

$s_{t}=\frac{N_{\text{mt}}(t+T_{\text{wt}})/N_{\text{mt}}(t)}{N_{\text{wt}}(t+T_{\text{wt}})/N_{\text{wt}}(t)}-1=2^{-(c_{1}/r_{\text{wt}})M_{\text{mt-wt}}}-1$. [8]

To estimate *c*_1_/*r*_wt_, we utilized the fact that a fitness cost of 3.2% was observed for a misfolded protein expressed at 0.1% of the proteome of the yeast cell . In other words, $-0.032=2^{-c_{1}\cdot(0.001\times12000\times5000)/r_{\text{wt}}}-1$, where 12000 is the total number of mRNA molecules per cell and 5000 is the average number of protein molecules made from each mRNA molecule per generation . Hence, *c*_1_/*r*_wt_ = 7.82×10^-7^.

We considered the relationship between elongation speed and translational error rate by following a recent study . Codon/tRNA selection on the ribosome contains two major discriminative steps, the initial selection and proofreading . For the initial selection, let us treat the ribosome as an enzyme (E), the ternary complex of aminoacylated tRNA∙eEF-1α∙GTP as a substrate (S), and the hydrolyzed ternary complex (aminoacylated tRNA∙eEF-1α∙GDP+Pi) as the product (P). The initial selection of cognate or noncognate tRNA can be described by

, [9]

where *k*_1_, *k*_-1_, and *k*_2_ are the rate constants of tRNA association with ribosome, dissociation with ribosome, and GTP hydrolysis on eEF-1α, respectively . The rate of P production is d[P]/d*t* = [SE]*k*_2_ = [S][E]*k*_1_*k*_2_/(*k*_-1_+*k*_2_) = [S][E]*K*, where *K = k*_1_*k*_2_/(*k*_-1_+*k*_2_). By definition, the elongation speed (*ν*) is the number of P produced per second per ribosome, or *ν* = (d[P]/d*t*)/[E]*=*[S]*K*. Now, let us consider cognate and noncognate substrates separately. Their concentrations are [S^c^] and [S^nc^], respectively, and their *K* values are $K^{\text{c}}=\frac{k_{1}^{\text{c}}k_{2}^{\text{c}}}{k_{-1}^{\text{c}}+k_{2}^{\text{c}}}$ and $K^{\text{nc}}=\frac{k_{1}^{\text{nc}}k_{2}^{\text{nc}}}{k_{-1}^{\text{nc}}+k_{2}^{\text{nc}}}$, respectively. Combining the cognate and noncognate reactions, we can calculate the error rate of the initial selection as

$a_{1}=\frac{[\text{S}^{\text{nc}}]K^{\text{nc}}}{[\text{S}^{\text{c}}]K^{\text{c}}+[\text{S}^{\text{nc}}]K^{\text{nc}}}=\frac{uK^{\text{nc}}}{K^{\text{c}}+uK^{\text{nc}}}$, [10]

where *u* = [S^nc^]/[S^c^]. The elongation speed is

$v=K^{\text{c}}[\text{S}^{\text{c}}]+K^{\text{nc}}[\text{S}^{\text{nc}}]=[\text{S}^{\text{c}}](K^{\text{c}}+uK^{\text{nc}})$. [11]

Let $d=(k_{1}^{\text{c}}/k_{1}^{\text{nc}})(k_{-1}^{\text{nc}}/k_{-1}^{\text{c}})(k_{2}^{\text{c}}/k_{2}^{\text{nc}})$, which can be assumed to be constant given the codon being translated (this is a crucial assumption; see text below Eq. [14]). It can be shown that

$\frac{1}{K^{\text{nc}}}=\frac{d}{K^{\text{c}}}-\frac{d-k_{1}^{\text{c}}/k_{1}^{\text{nc}}}{k_{1}^{\text{c}}}$. [12]

We can assume that the association of tRNA with ribosome is non-discriminative such that $k_{1}^{\text{c}}/k_{1}^{\text{nc}}=1$. Using Eqs. [10] and [12], we have

$K^{\text{c}}=k_{1}^{\text{c}}\frac{d-u/a_{1}+u}{d-1}$ and $uK^{\text{nc}}=\frac{a_{1}K^{\text{c}}}{1-a_{1}}$. [13]

Combining Eqs. [11] and [13], we have

$\frac{v}{[\text{S}^{\text{c}}]}=k_{1}^{c}\frac{u+d-u/a_{1}}{(d-1)(1-a_{1})}\approx k_{1}^{c}\frac{u+d-u/a_{1}}{d-1}$. [14]

As shown previously , *d* is determined entirely by the difference in standard free energy of the transition state for GTP hydrolysis between noncognate and cognate reactions. In a given cell for a given codon (say, CCC), *d* should not vary among the CCCs at different positions of a gene or in different genes. At least *d* is not expected to co-vary with *a*_1_. Although there is no reason to believe that the above condition is violated in reality, we would like to point out that if *d* co-varies with *a*_1_, our model may not hold. In a given cell for a given codon, *u* is a constant. Because cognate reactions are more efficient than noncognate reactions, *d* > 1 (see also empirically estimated *d* in the following paragraph). Thus, *ν*/[S^c^] is a linear function of 1/*a*_1_, translational accuracy. Eq. [14] clearly indicates the tradeoff between elongation speed and accuracy, and Fig. S1 illustrates the essence of the origin of this tradeoff with a simple analogy.

To estimate the slope and the intercept of the linear function in Eq. [14], we used the data collected from *E. coli* *in vitro* translation under various Mg^2+^ concentrations . It was found that when 2 and 4 mM extra Mg^2+^ was added, the reaction efficiency for the cognate AAA codon was 117 and 147 μM^-1^s^-1^, respectively . Under the same pair of environments, the total reaction efficiencies for nine near-cognate codons were ~0.6 μM^-1^s^-1^ and ~1.3 μM^-1^s^-1^, respectively . Here, the near-cognate codons each differ from the cognate codon by one nucleotide. We ignored noncognate codons that are not near-cognate because their reactions are expected to be much less efficient. Ignoring these codons renders our conclusion (that selection to minimize mistranslation trumps selection to minimize ribosome sequestration) conservative, because of the underestimation of the mistranslation rate and its fitness cost. The total reaction efficiency (ν/[S^c^]) in the pair of environments was then 117.6 and 148.3 μM^-1^s^-1^, respectively. Assuming similar reaction efficiencies for other tRNAs, the error rates under the same pair of environments were $0.6/(117+0.6)\approx5.1\times10^{-3}$and $1.3/(147+1.3)\approx8.8\times10^{-3}$, respectively. Solving Eq. [14] with these numbers, we obtained

$\frac{v}{[\text{S}^{\text{c}}]}=\frac{509.8-1/a_{1}}{2.669}$. [15]

Given that the physiological concentration of *E. coli* Lys tRNA ternary complex is ~0.2 μM , Eq. [15] can be transformed to $v=38.2-1/(13.3a_{1})$. For the proofreading step, detailed kinetic analysis on the relationship between error rate and speed is still missing. However, the selectivity (ratio between efficiencies of cognate and noncognate reactions) at the proofreading step has been estimated to be 6.5 to 15, respectively . Assuming that the average selectivity of this step is 10, the total error rate after the two steps is

$a=\frac{a_{1}\times1}{(1-a_{1})\times10+a_{1}\times1}=\frac{a_{1}}{10-9a_{1}}\approx\frac{a_{1}}{10}$. [16]

Combining Eq. [15] and [16], we obtained the relationship between the error rate *a* and elongation speed ν as

$\nu=38.2-0.00749/a$. [17]

When *v* = 10 or 30 codons per second, *a* = $2.67\times10^{-4}$ or $9.17\times10^{-4}$, which match the observed mistranslation rates . Combining Eqs. [6], [8] and [17] and assuming that the fraction of mistranslated proteins that are misfolded is *f*_t_ = 50%, we plotted *s*_t_ for various values of Δ*ν* and *E_g_* (Fig. 2B). Similar to *s_v_*, given Δ*ν*, the absolute value of *s*_t_ is greater when $\Delta v$occurs to a highly expressed gene than to a lowly expressed gene.

We then combined the above two fitness effects by $s=s_{v}+s_{\text{t}}$ to predict the theoretically optimal elongation speed (Fig. 2C). We found the fittest *Δν* to be -12.2 and -5.3 codons per second for genes with the highest (5000 mRNA molecules per cell) and lowest (1 mRNA molecule per cell) expressions considered, respectively. We inferred a negative correlation between the expression level of a gene and its optimal elongation speed (the dotted line in Fig. 2C). This prediction appears to be robust to variations of the parameters in the model, including gene length (200 to 600 codons), baseline elongation rate (15 to 30 codons per second), degradation rate (5×10^4^ to 1.5×10^5^ amino acids per 60 seconds), mean protein molecules produced per mRNA molecule (1000 to 9000), number of active ribosomes (1×10^5^ to 3×10^5^), total mRNA molecules per cell (6×10^3^ to 1.8×10^4^), and the fraction of mistranslated proteins that are misfolded (0.2-0.8) (Fig. 2D). It is worth pointing out here that, due to the complexity, we did not consider the loss-of-function effect of translational errors in our model. Because such errors are expected to have bigger effects on highly expressed genes than on lowly expressed genes , they would further reduce the optimal elongation speed for highly expressed genes, but would have a minimal impact on lowly expressed genes.

Our model is relatively simple, but it contains the essential elements pertaining to the hypothesis being tested and is constrained by the feasibility consideration because not all parameters would have known values in the literature. The model predicts a negative correlation between gene expression level and elongation speed, which is empirically supported. Based on the model, we estimated that the fitness effect of a single mutation altering the accuracy-efficiency tradeoff can greatly exceed the inverse of the effective population size of yeast, which is consistent with our hypothesis. Therefore, although the model is built under a general theoretical framework at the cost of specificity, its major conclusions appear sound.

**References**

1. Ghaemmaghami S, Huh WK, Bower K, Howson RW, Belle A, et al. (2003) Global analysis of protein expression in yeast. Nature 425: 737-741.

2. von der Haar T (2008) A quantitative estimation of the global translational activity in logarithmically growing yeast cells. BMC Syst Biol 2: 87.

3. Belle A, Tanay A, Bitincka L, Shamir R, O'Shea EK (2006) Quantification of protein half-lives in the budding yeast proteome. Proc Natl Acad Sci U S A 103: 13004-13009.

4. Gilchrist MA, Wagner A (2006) A model of protein translation including codon bias, nonsense errors, and ribosome recycling. J Theor Biol 239: 417-434.

5. Drummond DA, Wilke CO (2008) Mistranslation-induced protein misfolding as a dominant constraint on coding-sequence evolution. Cell 134: 341-352.

6. Geiler-Samerotte KA, Dion MF, Budnik BA, Wang SM, Hartl DL, et al. (2011) Misfolded proteins impose a dosage-dependent fitness cost and trigger a cytosolic unfolded protein response in yeast. Proc Natl Acad Sci U S A 108: 680-685.

7. Johansson M, Zhang J, Ehrenberg M (2012) Genetic code translation displays a linear trade-off between efficiency and accuracy of tRNA selection. Proc Natl Acad Sci U S A 109: 131-136.

8. Rodnina MV (2012) Quality control of mRNA decoding on the bacterial ribosome. Adv Protein Chem Struct Biol 86: 95-128.

9. Uemura S, Aitken CE, Korlach J, Flusberg BA, Turner SW, et al. (2010) Real-time tRNA transit on single translating ribosomes at codon resolution. Nature 464: 1012-1017.

10. Gromadski KB, Rodnina MV (2004) Kinetic determinants of high-fidelity tRNA discrimination on the ribosome. Mol Cell 13: 191-200.

11. Drummond DA, Wilke CO (2009) The evolutionary consequences of erroneous protein synthesis. Nat Rev Genet 10: 715-724.

12. Cherry JL (2010) Expression level, evolutionary rate, and the cost of expression. Genome Biol Evol 2: 757-769.

13. Gout JF, Kahn D, Duret L (2010) The relationship among gene expression, the evolution of gene dosage, and the rate of protein evolution. PLoS Genet 6: e1000944.
