# Supplementary material for: Codon-by-Codon Modulation of Translational Speed and Accuracy Via mRNA Folding
Source: PLoS Biol. 2014 Jul 22;12(7):e1001910. doi: 10.1371/journal.pbio.1001910 (PMC4106722; doi:10.1371/journal.pbio.1001910)
Supplement: Text S5 — Ribosome run-off data from mouse embryonic stem cells. (DOC) [file pbio.1001910.s009.doc]

**Text S5. Ribosome run-off data from mouse embryonic stem cells**

In their ribosome run-off experiment in mouse embryonic stem cells , Ingolia and colleagues first used harringtonine to stop translational initiation. A ribosome depleted region was created as the initiation was stopped and elongation continued. After different amounts of time, cycloheximide was added to stop elongation. The relationship between the length of the ribosome depleted region and the time between the applications of the two drugs provides an independent measure of the mean elongation speed for the ribosome depleted region . We downloaded the sequencing reads in the ribosome run-off experiment from NCBI GEO (accession numbers are GSM765297, GSM765294 and GSM765295 ), which were previously mapped to the mouse transcriptome by the authors. Reads with different lengths were assigned to specific ribosome A sites as previously described . Sequencing reads with non-unique mapping were handled as aforementioned for yeast ribosome profiling data. The ribosome density was then calculated for every five-codon window across the coding region of each gene. Finally, the ribosome densities across the whole gene were normalized such that the average ribosome density from codon 800 to 1000 equals 1, because this region showed no sign of ribosome depletion in the analyzed run-off time points .

As the sequencing coverage in the ribosome run-off data is rather low for most of the genes, we adapted the meta-gene approach used in the original study . Basically, genes were grouped according to either evolutionary conservation or expression level, and the ribosome footprints were pooled together within each group. To group by evolutionary conservation, we used the estimated mouse protein evolutionary conservation (i.e., the inverse of the evolutionary rate), and separated all genes with ribosome run-off data in all three time points into 30 equal-size groups. To group by gene expression level, we downloaded the expression levels in mouse embryonic stem cells (Table S2A in Ingolia et al. ), removed the 5% least expressed genes because their expression level estimates were likely to be unreliable, and then separated the remaining genes into 30 equal-size groups. Within each group, normalized ribosome densities as in Ingolia et al. were averaged among genes and then smoothed using the “lowess” function in R (provided by the “gplots” package, with the smoother span equal to 0.5). To outline the 3’ border of ribosome depleted regions, a cutoff of 0.9 was applied to the smoothed ribosome densities downstream of the first 15 codons. We then fit a linear model (function “lm” in R package “stats”) between the length of a ribosome depleted region and the corresponding run-off time, as previously described . The elongation speed was estimated by the slope of the fitted regression line, and its standard error was estimated by the standard error of the slope.

**References**

1. Ingolia NT, Lareau LF, Weissman JS (2011) Ribosome profiling of mouse embryonic stem cells reveals the complexity and dynamics of mammalian proteomes. Cell 147: 789-802.
