# Supplementary material for: Codon-by-Codon Modulation of Translational Speed and Accuracy Via mRNA Folding
Source: PLoS Biol. 2014 Jul 22;12(7):e1001910. doi: 10.1371/journal.pbio.1001910 (PMC4106722; doi:10.1371/journal.pbio.1001910)
Supplement: Text S6 — Selective strength on point mutations affecting mRNA secondary structures. (DOC) [file pbio.1001910.s010.doc]

**Text S6. Selective strength on point mutations affecting the mRNA secondary structure**

Suppose a mutation strengthens mRNA folding at *p* sites and consequently decreases the elongation speed for *p* codons by *q* fold. The averaged elongation speed for the mutant can be shown to be $v'=Lv/[L-p+pq]$, where *ν* is its original speed and *L* is the gene length in term of the number of codons. When *p* << *L*, we have

$\Delta v=\frac{p-pq}{L-(p-pq)}v\approx\frac{p-pq}{L}v$. [18]

To estimate *q*, we extracted the 100 most highly expressed genes with ribosome profiling data (for which measurements should be more accurate than for lowly expressed genes). We then compared the averaged ribosome density of sites whose PARS at offset +12 are among the highest 5% of all sites within a gene with the corresponding number for the lowest 5% of all sites within the gene, and observed a median decrease of 81% in ribosome density (Fig. S4N), resulting in *q* ≈ 5. For an average gene, if we further assume *p* = 1 codon, *L* = 400 codons, and *v* = 20 codon/s, we obtain $\Delta v=$-0.2 codon/s. This, according to the model described in Text S4, corresponds to a fitness effect of *s* = 0.007 and 1.5×10^-6^ for the most strongly and most weakly expressed genes, respectively. Given yeast’s effective population size of *N* ≈ 10 million , *Ns* >> 1. Thus, a mutation that alters the mRNA folding strength at a single site to impact translational speed and accuracy can be subject to substantial natural selection, especially when it occurs in highly expressed genes.

**References**

1. Wagner A (2005) Energy constraints on the evolution of gene expression. Mol Biol Evol 22: 1365-1374.
