# Supplementary material for: Codon-by-Codon Modulation of Translational Speed and Accuracy Via mRNA Folding
Source: PLoS Biol. 2014 Jul 22;12(7):e1001910. doi: 10.1371/journal.pbio.1001910 (PMC4106722; doi:10.1371/journal.pbio.1001910)
Supplement: Text S7 — Amino acid charge and elongation speed. (DOC) [file pbio.1001910.s011.doc]

**Text S7. Amino acid charge and elongation speed**

Charneski and Hurst reported that positively charged amino acids in the nascent peptide retards translational elongation of downstream codons , presumably through interaction with the negatively charged ribosome exit tunnel . But the nascent peptide often forms secondary structures within the exit tunnel , rendering the distance (in terms of the number of codons) between the positively charged residues and the downstream codon that is being stalled variable. Indeed, Charneski and Hurst found that positively charged amino acids only have a loosely defined ribosome stalling site . This property implies that although positively charged residues can be useful in modulating the elongation speed of individual codons, the distance between the positively charged amino acids and the downstream codon that is being modulated is expected to vary greatly, hindering the genome-wide detection of this signal. To exclude the possibility that our observation of the effect of mRNA secondary structure on elongation speed is a byproduct of that of positively charged amino acids, we repeated the analysis in Fig. 6 after removing all codons whose upstream 30 codons contain ≥ 5 positively charged amino acids. We found that the correlation between elongation speed and mRNA folding strength at offset +12 remains strong (ρ = -0.0905, binomial *P* < 10^-14^, Fig. S4L), suggesting that mRNA folding at offset +12 slows elongation independently of 5’ positively charged amino acids. Furthermore, within each gene, we computed the partial rank correlation between the evolutionary conservation of the encoded amino acid of a codon and its elongation speed after the control of the PARS score at offset +12. We found no significant difference between the resulting partial correlation and the corresponding random expectation (Fig. S4M), suggesting that mRNA secondary structures are the primary mechanism that modulates the tradeoff between translational accuracy and elongation speed. The fact that Charneski and Hurst examined only offsets 0 and +30, together with their requirement of PARS values (stretches of 30 codons whose average PARS value ≤0, immediately followed by a block of 31 codons whose average PARS value >0 ) explains why they failed to detect the effect of mRNA folding on the elongation speed.

**References**

1. Charneski CA, Hurst LD (2013) Positively charged residues are the major determinants of ribosomal velocity. PLoS Biol 11: e1001508.

2. Lu J, Deutsch C (2008) Electrostatics in the ribosomal tunnel modulate chain elongation rates. J Mol Biol 384: 73-86.

3. Fedyukina DV, Cavagnero S (2011) Protein folding at the exit tunnel. Annu Rev Biophys 40: 337-359.
