# Supplementary material for: Codon-by-Codon Modulation of Translational Speed and Accuracy Via mRNA Folding
Source: PLoS Biol. 2014 Jul 22;12(7):e1001910. doi: 10.1371/journal.pbio.1001910 (PMC4106722; doi:10.1371/journal.pbio.1001910)
Supplement: Text S8 — Quantifying mistranslation rates by dual luciferase assays. (DOC) [file pbio.1001910.s012.doc]

**Text S8. Quantifying mistranslation rates by dual luciferase assays**

Several versions of the plasmid pDB688 containing the Renilla-firefly fusion gene and different codons at codon 529 of the firefly segment were obtained from the P. J. Farabaugh lab, where this dual luciferase system was first used to measure translational error rates in yeast . The region of pDB688 containing the PGK promoter and the Renilla-firefly fusion gene was fused with kanMX6 from plasmid pFA6a (Addgene). Three versions of the 3’ sequence were used and their mRNA secondary structures predicted by RNAfold are listed as follows.

Wildtype -

AGAAAAATCAGAGAGATCCTCATAAAGGCCAAGAAGGGCGGAAAGTCCAAATTGTAA

......(((.....)))(((.....))).(((...((((.....))))...)))...

ΔG = -11.30 kcal/mol

Paired -

AGAAAGATCAGAGAGATCTTGATAAAAGCCAAGAAGGGCGGAAAATCCAAGCTCTAA

(..((((((.....))))))..)...........((((((((...)))..)))))..

ΔG = -15.52 kcal/mol

Unpaired -

AGAAAGATCAGAGAGATACTCATCAAAGCCAAGAAAGGAGGCAAGTCCAAGTTGTAA

.....(((..(((.....))))))...(((.........)))...............

ΔG = -6.94 kcal/mol

Note that all 3’ sequences generated by swapping synonymous codons and have +12 nucleotide paired have greater folding strengths than that of the wild-type. The 3’ end of the fusion gene was respectively replaced with the “paired” and “unpaired” 3’ sequences by PCR using designed primers. The modified fusion fragments were then used to replace the coding region of *HO* by homologous recombination in *S. cerevisiae* BY4741. All constructs were confirmed by DNA sequencing.

Dual luciferase assays in *S. cerevisiae* were performed as previously described . Briefly, transformed strains were grown at 30°C in YPD to OD600 = 1. Cells were collected by centrifugation at 4000 rpm, washed once, and then resuspended in 250 μL ice cold phosphate buffered saline (PBS). Cells were lysed by vortexing with glass beads for 10 min and clarified by centrifugation at 4000 rpm at 4°C. Using 10 μL of the supernatant, we assayed the firefly and Renilla luciferase activities using the Dual-Luciferase Reporter Assay System (Promega). Relative luminescence units (RLUs) were then measured for 10 sec using a GloMaxTM 96 Microplate Luminometer (Promega) according to the manufacturer’s instructions. All Renilla and firefly RLUs are between 10^2^ and 10^8^, within the linear dynamic range of the equipment. Three colonies of each construct were assayed in quadruplicates.

**References**

1. Kramer EB, Vallabhaneni H, Mayer LM, Farabaugh PJ (2010) A comprehensive analysis of translational missense errors in the yeast Saccharomyces cerevisiae. RNA 16: 1797-1808.
