# Supplementary material for: Codon-by-Codon Modulation of Translational Speed and Accuracy Via mRNA Folding
Source: PLoS Biol. 2014 Jul 22;12(7):e1001910. doi: 10.1371/journal.pbio.1001910 (PMC4106722; doi:10.1371/journal.pbio.1001910)
Supplement: Text S9 — Differential elongation speeds and balanced codon usage. (DOC) [file pbio.1001910.s013.doc]

**Text S9. Differential elongation speeds and balanced codon usage**

We previously demonstrated that, to minimize ribosome sequestration, transcriptomic synonymous codon usage should be proportional to the concentrations of their respective cognate tRNAs such that the cellular demand and supply of tRNAs are balanced . This model is unaffected by the present finding of variable elongation speeds within and between genes. This is because the time needed for recycling a tRNA is much longer than codon selection time . Specifically, it has been estimated that the physiological concentration of ternary complexes is only 4.3% of the total concentration of tRNAs , implying that an average tRNA spends at most ~4.3% of its time (and most likely only a fraction of that time) in active participation of translation; over ~95.7% of its time is spent in recycling. To estimate the time spent on translating fast and slow codons respectively, we utilized the data presented in Fig. 1B. We first removed genes whose protein or mRNA concentrations are within the top 10% or bottom 10% of all genes, because their translational initiation rate estimates may be unreliable (see Text S2). Using the remaining genes, we estimated that the average time spent on translating one codon is 59.2 units for the entire transcriptome. The corresponding time is 145.9 units for the 5% most highly expressed genes and 2.7 units for the 5% most weakly expressed genes. Thus, the mean time spent in translating a slow codon is 2.5 times the transcriptome average, while that spent in translating a fast codon is 5% the transcriptome average. Hence, the total time of participation of a tRNA in translation and recycling varies from 1.06 (= 0.043×2.5+0.957) unit for the slowest genes, to 1 unit for average genes and 0.96 (=0.043×0.05+0.957) unit for the fastest genes. Apparently, different elongation speeds hardly affect the supply of tRNAs for translation (Fig. S3E). Furthermore, as shown in Fig. 4A, there is no consistent elongation speed difference among synonymous codons of a given amino acid. As a result, the previously proposed model of balanced codon usage remains valid.

**References**

1. Qian W, Yang JR, Pearson NM, Maclean C, Zhang J (2012) Balanced codon usage optimizes eukaryotic translational efficiency. PLoS Genet 8: e1002603.
