# Supplementary material for: Codon-by-Codon Modulation of Translational Speed and Accuracy Via mRNA Folding
Source: PLoS Biol. 2014 Jul 22;12(7):e1001910. doi: 10.1371/journal.pbio.1001910 (PMC4106722; doi:10.1371/journal.pbio.1001910)
Supplement: Text S10 — Similarity in PARS between neighboring nucleotides. (DOC) [file pbio.1001910.s014.doc]

**Text S10. Similarity in PARS between neighboring nucleotides**

Within each gene, we calculated the rank correlation (ρ) in PARS between each nucleotide and the nucleotide immediately downstream of it. We found that more than 99% of the genes have ρ > 0. The average correlation for all genes is ρ = 0.512.
