# Supplementary material for: Codon-by-Codon Modulation of Translational Speed and Accuracy Via mRNA Folding
Source: PLoS Biol. 2014 Jul 22;12(7):e1001910. doi: 10.1371/journal.pbio.1001910 (PMC4106722; doi:10.1371/journal.pbio.1001910)
Supplement: Text S11 — Explanation of the simplified model in Figure S1A. (DOC) [file pbio.1001910.s015.doc]

**Text S11. Explanation of the simplified model in Fig. S1A**

Based on the reaction shown by Eq [9] in Text S4, the acceptance (*R_accept_*) to rejection (*R_reject_*) rate ratio for any tRNA is *k*_2_/*k*_-1_. Thus, for cognate (c) and noncognate (nc) tRNAs, it can be shown that

$\frac{R_{accept}^{c}}{R_{reject}^{c}}/\frac{R_{accept}^{nc}}{R_{reject}^{nc}}=\frac{k_{2}^{c}}{k_{-1}^{c}}\cdot\frac{k_{-1}^{nc}}{k_{2}^{nc}}$ [19]

Because (i) $d=(k_{1}^{\text{c}}/k_{1}^{\text{nc}})(k_{-1}^{\text{nc}}/k_{-1}^{\text{c}})(k_{2}^{\text{c}}/k_{2}^{\text{nc}})$can be assumed to be constant given the codon being translated , and (ii) the association of tRNA with ribosome can be assumed non-discriminative such that $k_{1}^{\text{c}}/k_{1}^{\text{nc}}=1$, the right side of Eq [19] is a constant. In other words, the ratio between the acceptance/rejection rate ratio of the cognate tRNA and that of the noncognate tRNA is a constant**.**

**References**

1. Johansson M, Zhang J, Ehrenberg M (2012) Genetic code translation displays a linear trade-off between efficiency and accuracy of tRNA selection. Proc Natl Acad Sci U S A 109: 131-136.

2. Rodnina MV (2012) Quality control of mRNA decoding on the bacterial ribosome. Adv Protein Chem Struct Biol 86: 95-128.
