# Supplementary material for: Codon-by-Codon Modulation of Translational Speed and Accuracy Via mRNA Folding
Source: PLoS Biol. 2014 Jul 22;12(7):e1001910. doi: 10.1371/journal.pbio.1001910 (PMC4106722; doi:10.1371/journal.pbio.1001910)
Supplement: Text S12 — Robustness of the effect of mRNA folding at offset +12 on elongation speed. (DOC) [file pbio.1001910.s016.doc]

**Text S12. Robustness of the effect of mRNA folding strength at offset +12 on elongation speed**

We considered the impacts of several potential errors in the ribosome footprint data and RNA folding strength data on our findings. First, when the ribosome-protected fragment ends with an A that could be aligned to the genome, it is impossible to determine whether the A was added as part of the polyA adaptor in the ribosome profiling library preparation, or was part of the actually ribosome-protected fragment of mRNA. To remove the potential effect of this uncertainty, we re-conducted the analysis shown in Fig. 6B using only the codons whose 28-nt ribosome footprints end with G or C (Fig. S4G). The analysis indicates that the uncertainty has no effect on our result.

Second, it is possible that ribosome-protected fragments can be aligned to multiple location of the yeast genome. We repeated Fig. 6B using only those reads that are uniquely aligned to the yeast genome, and found the effect of mRNA folding strength at offset +12 remains unaltered (Fig. S4H).

Third, the effect of RNA folding strength at off +12 on elongation speed can also be found when the RNA folding is measured at a relatively high temperature (Fig. S4I). This analysis used RNase V1-based per-nucleotide RNA folding strength measurement at 55˚C (GEO accession number GSE39680 , two replicates of RNA folding under 55˚C).

Fourth, to address the potential disparity in secondary structure between ribosome-free and ribosome-bound mRNAs, the secondary structures of mRNAs were also computationally predicted using RNAfold in the ViennaRNA package ([www.tbi.univie.ac.at/~ivo/RNA/](http://www.tbi.univie.ac.at/~ivo/RNA/)). We predicted mRNA secondary structures using sliding windows of 150 nucleotides with a step size of 10 nucleotides across the entire mRNA. The ribosome-bound mRNA folding strength of a nucleotide is defined by the number of sliding windows in which this nucleotide is paired with a downstream nucleotide, divided by the number of sliding windows that cover the nucleotide. The obtained results using these computationally predicted folding strengths (Fig. S4J) are similar to those in Fig. 6B.

Fifth, while this manuscript was under review, genome-wide data of yeast *in vivo* RNA folding strengths based on dimethyl sulphate (DMS) modification and next generation sequencing became available . We downloaded the number of DMS modification captured on each base pair of the yeast genome (GEO accession number GSE45803 , four replicates of *in vivo* yeast results) and assigned it to yeast genes (using SGD yeast genome R62, following the analytical pipeline of the original paper ). We defined the *in vivo* RNA folding strength of each nucleotide as the negated number of DMS modification captured on this nucleotide, because only single-stranded, unprotected nucleotides are sensitive to DMS treatment. We were able to reproduce the observation in Fig. 6B with the newly defined *in vivo* RNA folding strengths (Fig. S4K). Note that due to the nature of DMS modification, only codons with 28-nt ribosome footprints ending with A or C are used.

Sixth, the effect of (experimentally determined) mRNA folding strengths on elongation speed is independent of that of positively charged amino acids. We re-did the analysis of Fig. 6B with only those codons that have ≤ 5 positively charged amino acids in its preceding 30 codons. The observed peak at offset +12 does not change (Fig. S4L), excluding the possibility that our observation is due to the effect of positively charged amino acids. See also Text S7.

**References**

1. Barrett T, Wilhite SE, Ledoux P, Evangelista C, Kim IF, et al. (2013) NCBI GEO: archive for functional genomics data sets--update. Nucleic Acids Res 41: D991-995.

2. Wan Y, Qu K, Ouyang Z, Kertesz M, Li J, et al. (2012) Genome-wide measurement of RNA folding energies. Mol Cell 48: 169-181.

3. Hofacker IL, Fontana W, Stadler PF, Bonhoeffer S, Tacker M, et al. (1994) Fast folding and comparison of RNA secondary structures. Monatsh Chem 125: 167-188.

4. Lange SJ, Maticzka D, Mohl M, Gagnon JN, Brown CM, et al. (2012) Global or local? Predicting secondary structure and accessibility in mRNAs. Nucleic Acids Res 40: 5215-5226.

5. Rouskin S, Zubradt M, Washietl S, Kellis M, Weissman JS (2014) Genome-wide probing of RNA structure reveals active unfolding of mRNA structures in vivo. Nature 505: 701-705.
