# Supplementary material for: Codon-by-Codon Modulation of Translational Speed and Accuracy Via mRNA Folding
Source: PLoS Biol. 2014 Jul 22;12(7):e1001910. doi: 10.1371/journal.pbio.1001910 (PMC4106722; doi:10.1371/journal.pbio.1001910)
Supplement: Text S13 — Comparing the effect sizes on elongation speed by various factors. (DOC) [file pbio.1001910.s017.doc]

**Text S13. Comparing the effect sizes on elongation speed by various factors**

Due to the substantial per-codon variability of ribosomal densities, we pooled different codons together to estimate the effect size of each factor concerned. These analyses were first conducted within each gene to avoid estimating translation initiation rates. The median or mean value among all genes is then reported.

We calculated the ratio in average ribosome density between conserved (i.e., the encoded amino acid is invariant across six post-WGD fungal species) and unconserved codons within each gene. We then calculated the median (1.065) and mean (1.453) of the ratios for the 100 most highly expressed genes (for which ribosome densities are more accurately measured). The result shows that the elongation speed of conserved codons is on average 7% to 30% slower than the unconserved codons.

We calculated the ratio in average ribosome density between codons with strong RNA folding strengths at offset +12 and codons with weak RNA folding strengths at offset +12. The weak (or strong) folding is defined as nucleotides with the 5%, 10%, 20%, 30%, 40%, or 50% lowest (or highest) PARS within the gene. We then calculated the median of the ratios for the 100 most highly expressed genes (for which ribosome densities are more accurately measured). The results (Fig. S4N) show that a median increase in ribosomal density of 80% to 500% is observed for intermediate to extreme difference in PARS at offset +12.

To estimate the effect of wobble codons on ribosome density , we estimated the ratio in ribosome density between wobble and non-wobble codons with the yeast data . On average, the ribosomal density of A-site wobble codons is 1.18 times that of non-wobble codons. This number becomes 1.07 if P-site wobble codons are considered. Codons at other regions of the ribosome footprint are less influential according to the original paper . Thus, the effect of RNA folding strength at offset +12 on elongation speed is ~80%, whereas that of wobble codon usage is ~15%. Regarding the effect of amino acid identity on ribosome density , Fig. 1D of the original paper showed that the effect on ribosome density never exceeds 30% (log_2_ ratio <= 0.25).

Our analysis showed that the effect of mRNA folding strength at offset +12 on elongation speed is independent of that of positively charged amino acids (see Text S7). The effect size of positively charged amino acids is unlikely to exceed that of mRNA folding. It was shown in Fig. 5 of the original paper that the ribosomal density increases by ~50% in the less extreme cases where only 1 to 3 positively charged amino acids are present and it increases by ~100% in the extreme cases where 4 or more positively charged amino acids are present. We observed that ribosome density increases by ~80% when codons with the 50% lowest PARS are compared with those with the 50% highest PARS. Ribosome density increases by ~500% when codons with the 5% lowest PARS are compared with those with the 5% highest PARS. Hence, PARS at offset +12 has an effect on elongation speed that is at least comparable with if not larger than that of positively charged amino acids. Note, however, because positively charged amino acids likely exert their effect as a stretch of amino acids and affect elongation speed of multiple codons, whereas the mRNA folding strength likely imposes its effect on individual codons, the effect size of these two factors may not be directly comparable (see also Text S7).

**References**

1. Stadler M, Fire A (2011) Wobble base-pairing slows in vivo translation elongation in metazoans. RNA 17: 2063-2073.

2. Ingolia NT, Ghaemmaghami S, Newman JR, Weissman JS (2009) Genome-wide analysis in vivo of translation with nucleotide resolution using ribosome profiling. Science 324: 218-223.

3. Zinshteyn B, Gilbert WV (2013) Loss of a conserved tRNA anticodon modification perturbs cellular signaling. PLoS Genet 9: e1003675.

4. Charneski CA, Hurst LD (2013) Positively charged residues are the major determinants of ribosomal velocity. PLoS Biol 11: e1001508.
